# BET-Independent Murine Leukemia Virus Integration Is Retargeted *In Vivo* and Selects Distinct Genomic Elements for Lymphomagenesis

**DOI:** 10.1128/spectrum.01478-22

**Published:** 2022-07-19

**Authors:** Ivan Nombela, Martine Michiels, Dominique Van Looveren, Lukas Marcelis, Sara el Ashkar, Siska Van Belle, Anne Bruggemans, Thomas Tousseyn, Jürg Schwaller, Frauke Christ, Rik Gijsbers, Jan De Rijck, Zeger Debyser

**Affiliations:** a Laboratory for Molecular Virology and Gene Therapy, Department of Pharmaceutical and Pharmacological Sciences, KU Leuven, Leuven, Flanders, Belgium; b Department of Imaging and Pathology, Translational Cell and Tissue Research Lab, KU Leuven, Flanders, Belgium; c UZ Leuven, University Hospitals, Department of Pathology, KU Leuven, Flanders, Belgium; d Department of Biomedicine, University Children’s Hospital (UKBB), University of Basel, Basel, Switzerland; University of Manitoba

**Keywords:** BALB/c mouse, BET proteins, BRD4, integrase, integration site selection, lymphomagenesis, murine leukemia virus, retroviral integration

## Abstract

Moloney murine leukemia virus (MLV) infects BALB/c mice and induces T-cell lymphoma in mice. Retroviral integration is mediated by the interaction of the MLV integrase (IN) with members of the bromodomain and extraterminal motif (BET) protein family (BRD2, BRD3, and BRD4). The introduction of the W390A mutation into MLV IN abolishes the BET interaction. Here, we compared the replication of W390A MLV to that of wild-type (WT) MLV in adult BALB/c mice to study the role of BET proteins in replication, integration, and tumorigenesis *in vivo*. Comparing WT and W390A MLV infections revealed similar viral loads in the blood, thymus, and spleen cells. Interestingly, W390A MLV integration was retargeted away from GC-enriched genomic regions. However, both WT MLV- and W390A MLV-infected mice developed T-cell lymphoma after similar latencies represented by an enlarged thymus and spleen and multiorgan tumor infiltration. Integration site sequencing from splenic tumor cells revealed clonal expansion in all WT MLV- and W390A MLV-infected mice. However, the integration profiles of W390A MLV and WT MLV differed significantly. Integrations were enriched in enhancers and promoters, but compared to the WT, W390A MLV integrated less frequently into enhancers and more frequently into oncogene bodies such as *Notch1* and *Ppp1r16b*. We conclude that host factors direct MLV *in vivo* integration site selection. Although BET proteins target WT MLV integration preferentially toward enhancers and promoters, insertional lymphomagenesis can occur independently from BET, likely due to the intrinsically strong enhancer/promoter of the MLV long terminal repeat (LTR).

**IMPORTANCE** In this study, we have shown that the *in vivo* replication of murine leukemia virus happens independently of BET proteins, which are key host determinants involved in retroviral integration site selection. This finding opens a new research line in the discovery of alternative viral or host factors that may complement the dominant host factor. In addition, our results show that BET-independent murine leukemia virus uncouples insertional mutagenesis from gene enhancers, although lymphomagenesis still occurs despite the lack of an interaction with BET proteins. Our findings also have implications for the engineering of BET-independent MLV-based vectors for gene therapy, which may not be a safe alternative.

## INTRODUCTION

The integration of the retroviral genome into the host genome is a key step in the replication cycle of retroviruses. Several studies have pointed out that integration site selection of retroviruses is targeted and not a random event (1). This event is specific to distinct retroviral families (2). For instance, human immunodeficiency virus type 1 (HIV-1) integration is driven toward active transcriptional units, while murine leukemia virus (MLV) integration is directed toward active enhancers and promoters (3–6). The main viral determinant coordinating integration is the integrase (IN). The biochemical reactions of retroviral integration are well understood. After reverse transcription, IN cleaves specific phosphodiester bonds near the viral DNA ends during the 3′-end processing reaction. Next, IN uses the resulting viral DNA 3′-OH groups during strand transfer to cut target DNA. Simultaneously, IN joins each viral DNA end to target DNA 5′-end phosphates. Both reactions proceed via the direct transesterification of phosphodiester bonds (7).

Lens epithelium-derived growth factor (LEDGF)/p75 is a direct binding partner of HIV-1 IN and specific for lentivirus integration (8). It tethers the viral preintegration complex (PIC) to the chromatin (9–12). The tethering mechanism of LEDGF/p75 requires the direct interaction of the integrase binding domain (IBD) at the C terminus with HIV-1 IN and the recognition of the H3K36m2/3 mark on nucleosomes through the N-terminal PWWP domain (13). Small-molecule inhibitors of the interaction between HIV-1 integrase and LEDGF/p75 have been developed and termed LEDGINs (14). The addition of LEDGINs during HIV infection not only reduces integration but also targets residual provirus away from H3K36me2/3 (13, 15). The residual provirus, targeted away from its preferential chromatin landscape, is transcriptionally less active, even after reactivation. It appears that retroviruses evolved to adopt epigenetic readers to find preferential integration sites associated with active transcription and/or latency (16).

In the case of MLV, proteins of the bromodomain and extraterminal domain (BET) family, which include BRD2, BRD3, and BRD4, were identified as binding partners of MLV IN and proposed to tether the MLV PIC to transcription start sites (TSSs) and enhancers (17–20). BET proteins are composed of two conserved N-terminal bromodomains (bromodomain 1 [BD1] and BD2), an extraterminal (ET) domain, and a C-terminal domain (CTD), which is present only in BRD4 (21). BD1 and BD2 are regions with hydrophobic amino acids able to recognize acetylated H3 and H4 tails (22) and act as chromatin readers. The ET domain associates with a variety of viral and cellular proteins, including transcription factors, chromatin-modifying factors, and histone-modifying enzymes (23). The CTD is necessary for the recruitment of positive transcription elongation factor b (P-TEFb) by the transcriptional complex (24). The MLV IN-BET interaction is mediated through the ET domain of BET proteins and the C-terminal domain of MLV IN (25) and results in the tethering of MLV IN to nucleosomes (26). Amino acids located between positions 390 and 405 of the MLV IN sequence define a conserved domain in gammaretroviruses involved in the interaction with BRD4 (25). A single substitution of tryptophan for alanine at position 390 (W390A) in this domain is sufficient to prevent the interaction with BRD2, BRD3, and BRD4 (19). BET proteins promote the efficient integration of MLV into the host genome (20). However, BET-independent MLV replicates with viral titers similar to those of wild-type (WT) MLV in cell culture (27), although its integration profile differs from that of the WT toward a more random distribution away from transcriptionally active genomic regions and transcriptional start sites (28). Therefore, the extent to which BET proteins are key factors for the replication and integration of MLV is still a matter of debate. In addition to this, retroviral p12 may also play a role in tethering MLV PICs to mitotic chromosomes to facilitate integration (29).

MLV has been studied as one of the prototype oncogenic animal retroviruses since the 1950s when it was noticed that leukemia could be transmitted to newborn mice by an unknown agent (30). This horizontal transmission occurs primarily through milk ingestion, while transmission by the venereal route is less common (31). MLV induces either lymphoblastic leukemia (31) or lymphoma in mice, and an enlarged thymus, spleen, or lymph node is present in infected adults (32). Since MLV can infect several B or T cells, either leukemia or lymphoma of these cell lineages or their corresponding immature lineages can be induced (33).

Upon integration, MLV can modify mouse gene expression by insertional mutagenesis (34). This phenomenon is characterized by the deregulation of gene expression at the transcriptional or posttranscriptional level (35, 36) and requires viral promoter and enhancer elements located in the U3 region of the long terminal repeats (LTRs) of the MLV genome. Therefore, provirus insertion at the start of the gene may enhance the transcription of the target gene from the retroviral promoter. Alternatively, viral integration into the central part or close to the 3′ end of a gene may produce a truncated form of the native protein, which can abort its regulatory control. Screening for MLV insertional mutagenesis in mouse models has been instrumental in identifying human oncogenes (37–39). Genes identified as targets for retroviral integration that also act as oncogenes are *Notch1*, *Pim1*, *Myc*, *Myb*, *Gfi*, and *Pvt1*, among others (40).

Many recent studies on the integration site selection of MLV were performed in the context of MLV-based vectors for human gene therapy (41–43). MLV-based vectors have been successfully used in clinical trials for patients with adenosine deaminase deficiency (44). However, serious safety concerns were raised when patients with X-linked severe combined immunodeficiency disease (X-SCID) developed T-cell leukemia after treatment with MLV-based viral vectors by the activation of the LMO2 oncogene (45–47). Several generations of viral vectors have been designed in order to increase the biosafety of retroviral therapy. A “second” generation is characterized by a self-inactivating (SIN) vector ensuring a deletion in the 3′ LTR after reverse transcription that abolishes the LTR promoter activity (48). Most recently, a “third” generation of retroviral vectors was proposed. This generation is characterized by a mutation in MLV IN abolishing the interaction with BET proteins to achieve an integration profile targeted away from the TSSs of oncogenes under *in vitro* conditions (27, 28, 49). Still, our knowledge of the impact of integration site selection on oncogenesis *in vivo* is poor.

In fact, the role of retroviral host factors such as LEDGF/p75 and BET in retroviral pathogenesis in their natural host has not been unambiguously demonstrated. HIV replication in humans in the absence of LEDGF/p75 has not been investigated. Upcoming clinical trials with LEDGINs as HIV treatments or cures may provide this information in the future (50). Loyola et al. studied the pathogenesis of a C-terminally deleted MLV clone in a tumorigenesis model (51). That previous study was associated with some recombination events with endogenous retroviruses.

Here, we explored the role of the BET-MLV IN interaction in retroviral replication, integration site selection, and lymphomagenesis *in vivo*. In contrast to a C-terminal truncation, the site-specific W390A mutant replicated to the same levels as those of wild-type MLV. Mice infected with W390A MLV developed T-cell lymphoma, to an extent similar to that in mice infected with WT MLV. The W390A mutation targeted integration away from enhancers but increased integration into the bodies of known oncogenes. Our observations indicate that the loss of the BET-MLV IN interaction redirects viral integration sites but does not abrogate MLV-mediated lymphomagenesis. In addition, our work also clarifies the relative contribution of viral integration at enhancers versus promoters or gene bodies to MLV-induced insertional mutagenesis.

## RESULTS

### Efficient BET-independent *in vitro* replication of the W390A MLV mutant.

To investigate the role of BET proteins during MLV replication *in vivo*, we generated two BET-independent Moloney MLV molecular clones: W390A and a C-terminal truncation mutant. Previous studies revealed that IN W390A is essential for the interaction of IN with BET proteins and, as such, affects the integration site pattern of MLV (27, 28). In addition, it was shown previously that this mutation does not hamper the transduction efficiency of MLV-derived viral particles (46). However, its effect on multiple-round viral replication was not assessed previously. First, a codon-optimized sequence duplicating the overlapping sequence at the protein level was inserted in front of the *env* start codon in molecular clone p63.2. We created WT p63.2 to introduce the W390A mutation a single time into MLV IN. Otherwise, the introduction of this mutation into parental p63.2 would have caused selective pressure to revert this mutation due to the presence of the wild-type codon in the overlapping Env gene (see Fig. S1A in the supplemental material). We also generated ΔC p63.2, carrying a deletion in the C-terminal domain of MLV IN between positions 382 and 408 (Fig. S2) (52).

HEK 293T cells were transfected with the plasmids p63.2, WT p63.2, W390A p63.2, and ΔC p63.2 to compare the levels of production of viral particles (Fig. S1B). The reverse transcriptase (RT) activity in the supernatant was measured at 48 h posttransfection. Both WT p63.2 and W390A p63.2 were at least as efficient as the parental p63.2 viral clone in the production of viral particles (Fig. S1C). However, the truncation of the C-terminal tail (p63.2 ΔC) resulted in a 3-fold decrease in RT activity in comparison with that of the parental p63.2 clone (Fig. S1C). Next, NIH 3T3 cells were infected with equal RT units (RTU) of MLV 63.2, WT 63.2, W390A MLV, and ΔC MLV molecular clones (Fig. S1B). In contrast to the other variants, the ΔC p63.2 clone did not efficiently replicate in NIH 3T3 cells (Fig. S1D). Since the C-terminal deletion hampers viral production and replication, this viral clone was omitted from further experiments.

### W390A MLV replicates at wild-type levels in BALB/c mice.

**FIG 1:**
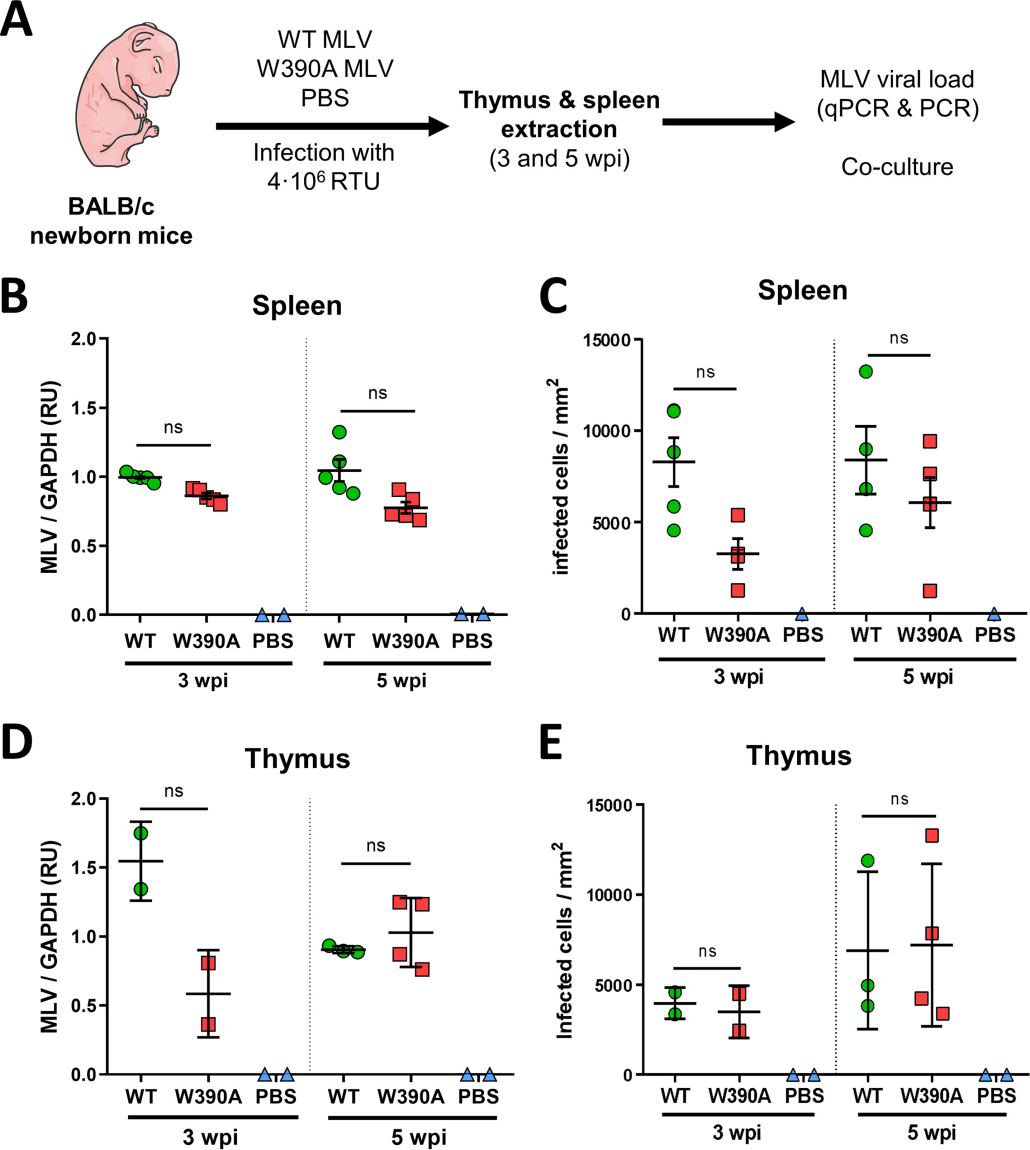
*In vivo* replication of WT MLV and W390A MLV in murine tissue. (A) Schematic representation of the workflow to evaluate the *in vivo* infectivity of WT MLV and W390A MLV. Newborn mice were infected by intraperitoneal injection with 4 × 10^5^ RTU of WT MLV or W390A MLV 1 day after birth. Blood was drawn between 80 and 90 days after injection. Extracts of the spleen and thymus were made at 3 and 5 weeks postinfection. (B) MLV loads in spleen cells from mice infected with WT MLV (*n* = 5) or W390A MLV (*n* = 5) or injected with PBS (*n* = 2) at 3 and 5 weeks postinfection. The viral load was measured by RT-qPCR of MLV IN relative to GAPDH levels and represented as relative units (RU). (C) Number of infected NIH 3T3 cells after 1 day of coculture with cells from spleens of mice infected with WT MLV (*n* = 4) or W390A MLV (*n* = 4) or injected with PBS (*n* = 2) at 3 and 5 weeks postinfection. (D) MLV loads in thymus cells from mice infected with WT MLV (3 wpi, *n* = 2; 5 wpi, *n* = 3) or W390A MLV (3 wpi, *n* = 2; 5 wpi, *n* = 4) or injected with PBS (*n* = 2) at 3 and 5 weeks postinfection. The viral load was measured by RT-qPCR of MLV IN relative to GAPDH levels. (E) Number of infected NIH 3T3 cells after 1 day of coculture with cells from spleens of mice infected with WT MLV (3 wpi, *n* = 2; 5 wpi, *n* = 3) or W390A MLV (3 wpi, *n* = 2; 5 wpi, *n* = 4) or injected with PBS (*n* = 2) at 3 and 5 weeks postinfection. No statistically significant difference was found using a Kruskal-Wallis test (B to E). ns, not significant.

Considering that WT MLV and W390A MLV can replicate to the same extent *in vitro*, we evaluated their infectivity *in vivo* next. Newborn BALB/c mice were injected with 4 × 10^6^ RTU of WT or W390A MLV or 50 μL of phosphate-buffered saline (PBS), as indicated in Fig. 1A. As a measure of MLV replication, MLV IN RNA expression was analyzed in spleen and thymus samples by reverse transcription-quantitative PCR (RT-qPCR). MLV IN RNA levels in spleen and thymus samples were similar after infection with WT MLV and W390A MLV, although a trend toward a lower viral load was seen with W390A MLV in the thymus at 3 weeks postinfection (wpi) (Fig. 1B and D). Additionally, *in vivo* infectivity was assessed by coculture of NIH 3T3 cells with spleen or thymus cells extracted at 3 or 5 wpi from mice infected with WT or W390A MLV. No statistically significant difference was found in the numbers of infected cells in coculture with the spleen or thymus of either WT MLV- or W390A MLV-infected mice, although a trend toward lower infection of spleen cells infected with W390A MLV was seen at 3 wpi (Fig. 1C and E). To exclude revertants of W390A MLV, we sequenced part of the MLV IN gene in 6 mice infected with parental 63.2 MLV, WT MLV, or W390A MLV at 12 weeks postinfection (Fig. S3). Although some synonymous and nonsynonymous base changes were detected, the alanine codon GCA remained intact.

### W390A MLV retargets viral integration.

**FIG 2:**
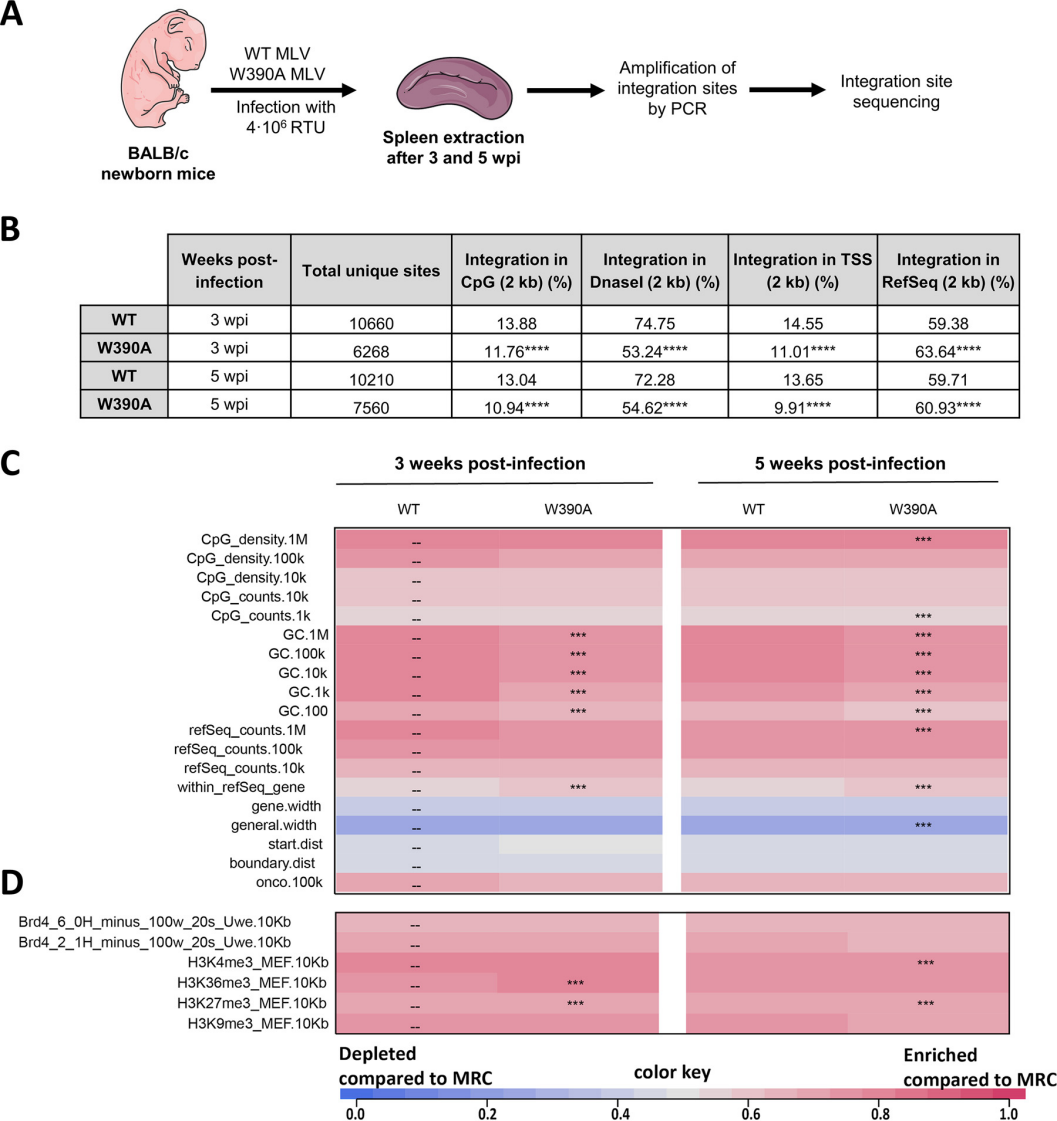
Integration site analysis of WT MLV and W390A MLV in infected mice. (A) Schematic representation of the workflow to determine the integration sites and the epigenetic features of WT MLV and W390A MLV integrants *in vivo*. (B) Genomic distribution of MLV integration sites obtained from spleen cells of mice infected with either WT MLV or W390A MLV at 3 and 5 wpi. Integration percentages for 2-kb windows around CpG-rich island midpoints, DNase I-hypersensitive sites (DHSs), transcription start sites (TSSs), and RefSeq genes are given. Statistical analysis was done using a chi-squared test comparing integrations from WT MLV-infected mice with integrations from W390A MLV-infected mice (*P* ≤ 0.0001). (C and D) Heatmaps depicting the relationship between integration site frequency and different genomic (C) or epigenetic (D) features within a 10-kb interval in splenic cells from mice infected with either WT MLV or W390A MLV. The data shown are based on pooled samples from 3 mice infected with either WT MLV or W390A MLV. Features analyzed are shown to the left of the corresponding row of the heatmap. Colors indicate whether a particular feature is disfavored (blue) or favored (red) for the integration of the respective data sets relative to their computer-generated matched random controls (MRCs), as detailed in the color scale at the bottom. Significant departures from WT MLV integration sites in splenic cells are based on a Wald test, referred to as the χ^2^ distribution.

Next, we analyzed integration preferences relative to a set of genomic and epigenetic features. W390A IN is known to shift the integration site preference in cell culture (27, 28). In fact, previous *in vitro* results showed a BET-independent integration profile characterized by lower levels of integration into transcription start sites (TSSs) of RefSeq genes, CpG islands, and DNase I-hypersensitive sites (DHSs) (28). Integration sites in spleen samples of mice infected with WT or W390A MLV were analyzed at 3 and 5 weeks postinfection (Fig. 2A). Fig. 2B summarizes the distribution of integration sites for several genomic features in a 2-kb window. A total of 6,000 to 10,000 integration sites were obtained under each condition by pooling genomic DNA from spleen cells of three mice infected with either WT or W390A MLV. The presence of W390A significantly shifted integration away from regions enriched in CpG islands, DNase I-hypersensitive sites, GC-enriched regions, and TSSs (Fig. 2C and D), and integration occurred more frequently in positions located within RefSeq genes at both tested times postinfection (Fig. 2B). With respect to epigenetic features, W390A MLV integration shifted toward histone marks associated with transcriptionally active and open chromatin, such as H3K36me3 (Fig. 2D), often related with gene bodies (53). In general, these data demonstrate that the *in vivo* integration site preference of BET-independent MLV in mouse spleen cells differs from that of WT MLV, although the extent of retargeting of the virus away from CpG and TSSs (but not from DHSs) was less than that observed with retroviral vectors in culture (27, 28); BET-independent MLV was retargeted away from all these genomic features, pointing to the role of BET in determining MLV integration sites *in vivo*. Moreover, MLV replication *in vivo* may negatively select for some integration sites due to cytopathogenicity, in contrast to single-round MLV vectors.

### Both WT MLV and W390A MLV induce lymphoma in infected mice.

**FIG 3:**
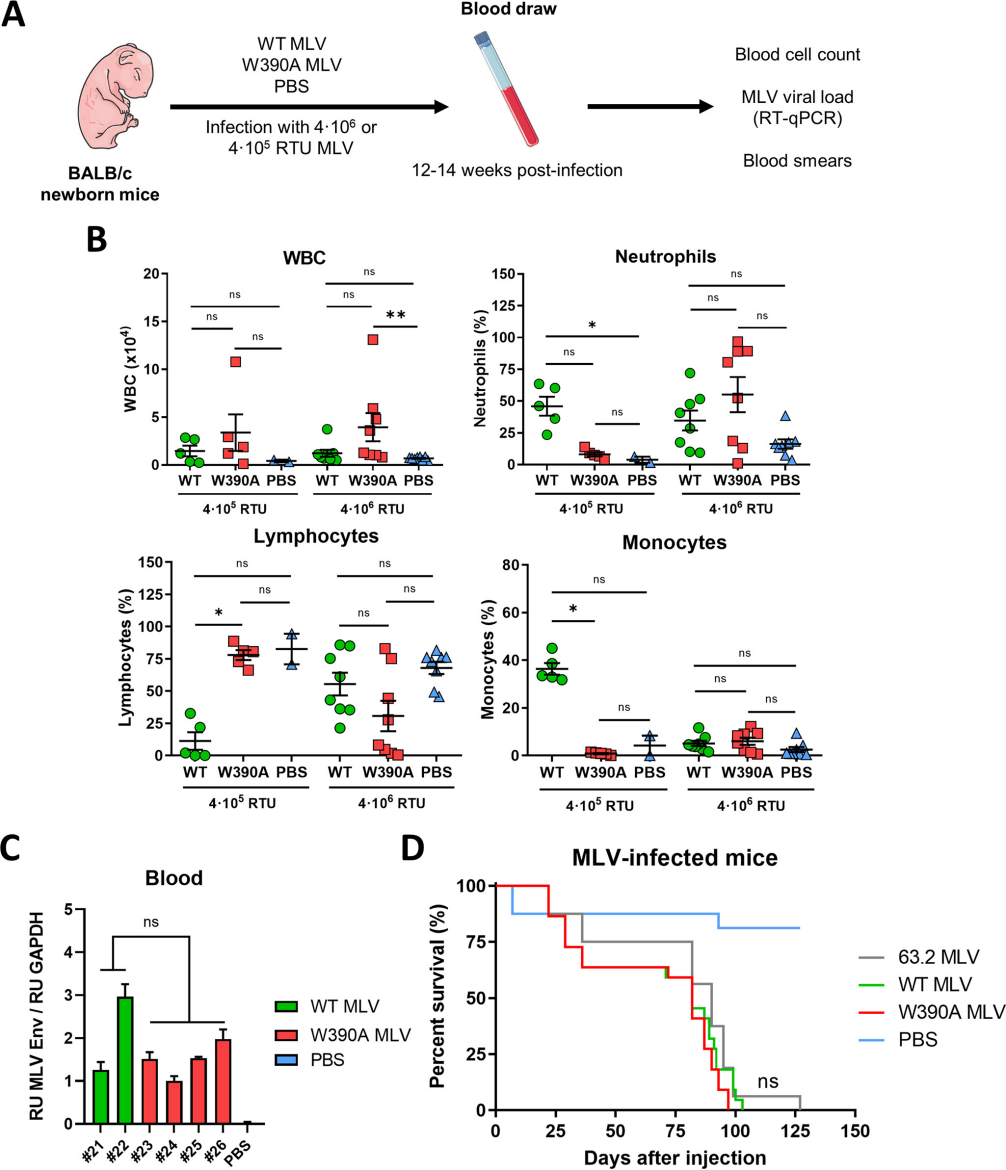
Blood cell counts in WT MLV- and W390A MLV-infected mice. (A) Schematic representation of the workflow to evaluate the long-term effect of WT MLV and W390A MLV replication on blood cell numbers. Newborn mice were infected by intraperitoneal injection with 4 × 10^5^ RTU or 4 × 10^6^ RTU of either WT MLV or W390A MLV 1 day after birth. Blood was drawn between 80 and 90 days after injection. PBS was used as a negative control. (B) Number of WBCs in blood samples of mice infected with WT MLV (4 × 10^5^ RTU, *n* = 5; 4 × 10^6^ RTU, *n* = 8) or W390A MLV (4 × 10^5^ RTU, *n* = 5; 4 × 10^6^ RTU, *n* = 8) or injected with PBS (*n* = 2 and 8 for comparisons with 4 × 10^5^ RTU and 4 × 10^6^ RTU, respectively). Data correspond to the number of WBCs in 1 μL of blood. Percentages of neutrophils, lymphocytes, and monocytes in WBC samples are given for mice infected with WT MLV or W390A MLV. PBS injection was used as a negative control. Horizontal lines indicate means ± standard deviations (SD), while dots represent individual values. Statistical significance was analyzed using a Kruskal-Wallis test. (C) MLV loads measured by RT-quantitative PCR of the MLV Env gene normalized to GAPDH levels in samples of whole blood from mice infected with WT MLV or W390A MLV, taken at 21 days postinfection. Each bar represents an individual mouse. Standard deviations were calculated from technical duplicates. (D) Survival rates of mice infected with 63.2 MLV, WT MLV, or W390A MLV. Newborn mice were infected with 4 × 10^6^ RTU of each molecular clone diluted in a total volume of 50 μL PBS. Kaplan-Meier plots display the survival of mice infected with 63.2 MLV (*n* = 16), WT MLV (*n* = 22), or W390A MLV (*n* = 22). PBS was used as a negative control (*n* = 16). Each vertical step in the curve indicates one or more deaths. No statistically significant difference was found among 63.2 MLV, WT MLV, and W390A MLV using a Gehan-Breslow-Wilcoxon test.

Virus-induced hematological malignancies are characterized by increased white blood cell (WBC) proliferation (54) and tumor infiltration into several organs and tissues, including the lymph nodes, spleen, and thymus (55). For this reason, we evaluated the pathology in blood, spleen, and thymus samples from mice infected with either WT or W390A MLV at 12 to 14 weeks postinfection. First, the proliferation of WBCs was assessed as described in the legend of Fig. 3A. Mice injected with 4 × 10^5^ RTU MLV (low dose) displayed an increased WBC count (1.46 × 10^4^ ± 1.24 × 10^4^ WBCs for WT MLV and 3.38 × 10^4^ ± 4.26 × 10^4^ WBCs for W390A MLV) compared to those in mice injected with PBS (0.42 × 10^4^ ± 0.12 × 10^4^ WBCs), although this difference was not statistically significant (Fig. 3B). Similarly, no statistically significant difference was found between mice infected with WT MLV and those infected with W390A MLV. Mice infected with 4 × 10^6^ RTU MLV (high dose) displayed an increased WBC count (1.22 × 10^4^ ± 1.04 × 10^4^ WBCs for WT MLV and 3.94 × 10^4^ ± 4.18 × 10^4^ WBCs for W390A MLV). Here, the increase in WBCs was statistically significant in W390A MLV-infected mice (*P* = 0.0044). After low-dose infection, WT MLV induced an increase in the relative number of neutrophils and monocytes and a decrease in the number of lymphocytes, which was not observed with W390A MLV. This phenotype was not seen at the high dose (Fig. 3B). At the high dose, the percentages of monocytes, lymphocytes, and neutrophils were similar among mice infected with WT MLV and W390A MLV and those injected with PBS (Fig. 3B). A summary of the blood cell counts and statistics can be found in Table S2.

The viral load in whole blood of mice infected with WT MLV (*n* = 2) or W390A MLV (*n* = 4) was measured by RT-qPCR to detect Env gene mRNA at 21 days postinfection. The statistical analysis did not reveal any significant difference (Fig. 3C).

We next compared the rates of survival of mice infected with 4 × 10^6^ RTU WT MLV and W390A MLV. In this experiment, newborn mice were also infected with parental 63.2 MLV. An overview of the different infection experiments is shown in Table S3, while Fig. 3D shows the pooled data. Mice infected with the distinct MLV clones showed similar survival rates (Fig. 3D). Although the median survival time was slightly higher for mice infected with MLV 63.2 (90 days) than for those infected with either WT MLV or W390A MLV (82 days), no statistically significant difference was found between these MLV clones. A detailed heatmap of mouse survival, including statistical analysis, can be found in Table S3. Here, mice infected with W390A MLV display a trend toward reduced survival.

### Histopathology of mouse spleen and thymus after MLV infection.

**FIG 4:**
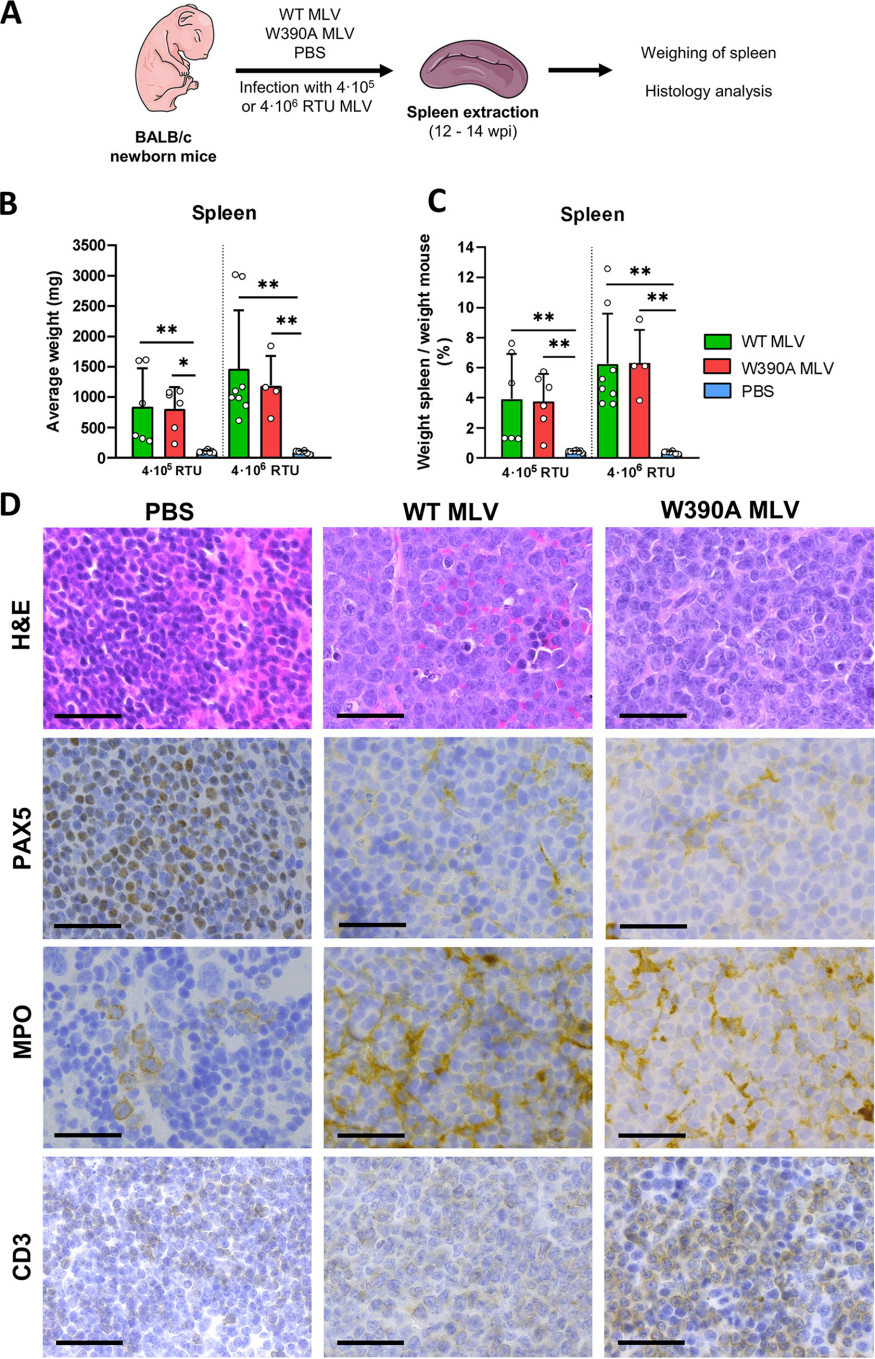
Spleen pathology in WT MLV- and W390A MLV-infected mice. (A) Schematic representation of the workflow to analyze the pathology in spleens from WT MLV- and W390A MLV-infected mice. Spleens were sampled between 80 and 90 days after infection. (B) Average weight of the spleens of mice infected with 4 × 10^6^ RTU of WT MLV (4 × 10^5^ RTU, *n* = 6; 4 × 10^6^ RTU, *n* = 9) or W390A MLV (4 × 10^5^ RTU, *n* = 6; 4 × 10^6^ RTU, *n* = 4) or injected with PBS (*n* = 8 and 6 for comparisons with 4 × 10^5^ RTU and 4 × 10^6^ RTU, respectively) as negative controls. Bars show means ± SD, while dots represent individual values. Statistically significant differences were analyzed using a Kruskal-Wallis test. (C) Percentage of the weight of the spleen in relation to the body mass of mice infected with WT MLV (4 × 10^5^ RTU, *n* = 6; 4 × 10^6^ RTU, *n* = 9) or W390A MLV (4 × 10^5^ RTU, *n* = 6; 4 × 10^6^ RTU, *n* = 4) or injected with PBS (*n* = 8 and 6 for comparisons with 4 × 10^5^ RTU and 4 × 10^6^ RTU, respectively). Bars show means ± SD, while dots represent individual values. Statistics were done using the Kruskal-Wallis test. (D) H&E, PAX-5, MPO, and CD3 staining of histological sections of spleens. Mice were infected with 4 × 10^6^ RTU of WT MLV or W390A MLV or injected with PBS as described above. Representative images are shown. Black arrows point to mitotic figures. PBS was used as a negative control. Bars, 20 μm.

Next, we analyzed the pathology of the spleen and thymus of mice infected with either WT or W390A MLV (Fig. 4A). At 3 and 5 weeks postinfection, prior to the development of lymphoma, spleens from mice infected with WT or W390A MLV were not enlarged (Fig. S4). In contrast, at late stages of the disease (12 to 14 weeks postinfection), the spleens of mice infected with either WT or W390A MLV were enlarged 20-fold compared to spleens from healthy mice injected with PBS (Fig. 4B and C). No statistically significant difference was found between WT MLV and W390A MLV.

Hematoxylin and eosin (H&E) staining of spleens from mice infected with WT MLV or W390A MLV revealed an expansive white pulp with a malignant lymphoid population characterized by large, irregular nuclei and scant cytoplasm (Fig. 4D). The irregular nuclei displayed open chromatin and variably prominent nucleoli. Moreover, frequent mitotic figures were present in the spleens of mice infected with WT or W390A MLV. As opposed to these findings, spleens from mice injected with PBS displayed normal white and red pulp. In these healthy mice, the white pulp consists of small lymphocytes with rounded nuclei (Fig. 4D). We also determined the lineage contribution of the tumor cells by staining for PAX-5 (B cells), myeloperoxidase (MPO) (myeloid cells), and CD3 (T cells) (56–58). PAX-5 staining of samples from mice injected with PBS depicted normal nuclear staining of B cells in a lymphoid follicle (Fig. 4D), while MPO staining identified mainly myeloid precursors in the red pulp (Fig. 4D). PAX-5 and MPO staining of diseased mice showed nuclear and cytoplasmic staining, respectively, and the malignant population scored negative for either marker. In contrast to the markers MPO and PAX-5, CD3 staining of spleen samples from WT MLV-infected mice revealed relatively weak but extended expression across the samples, while in the spleen samples from mice infected with W390A MLV, staining was stronger. Pathology results were compatible with the development of T-cell lymphoma in mice infected with either WT MLV or W390A MLV.

Tumor infiltration was also observed in lung, liver, and kidney tissues from mice infected with WT MLV or W390A MLV (Fig. S5). Overall, these results demonstrate that mutant W390A MLV still induces T-cell lymphoma. No overt pathological differences were evidenced between BET-dependent (WT) MLV and BET-independent (W390A) MLV, although stronger CD3 staining was evidenced with BET-independent MLV.

### Both WT MLV- and W390A MLV-induced lymphomas display clonal expansion yet different integration profiles.

**FIG 5:**
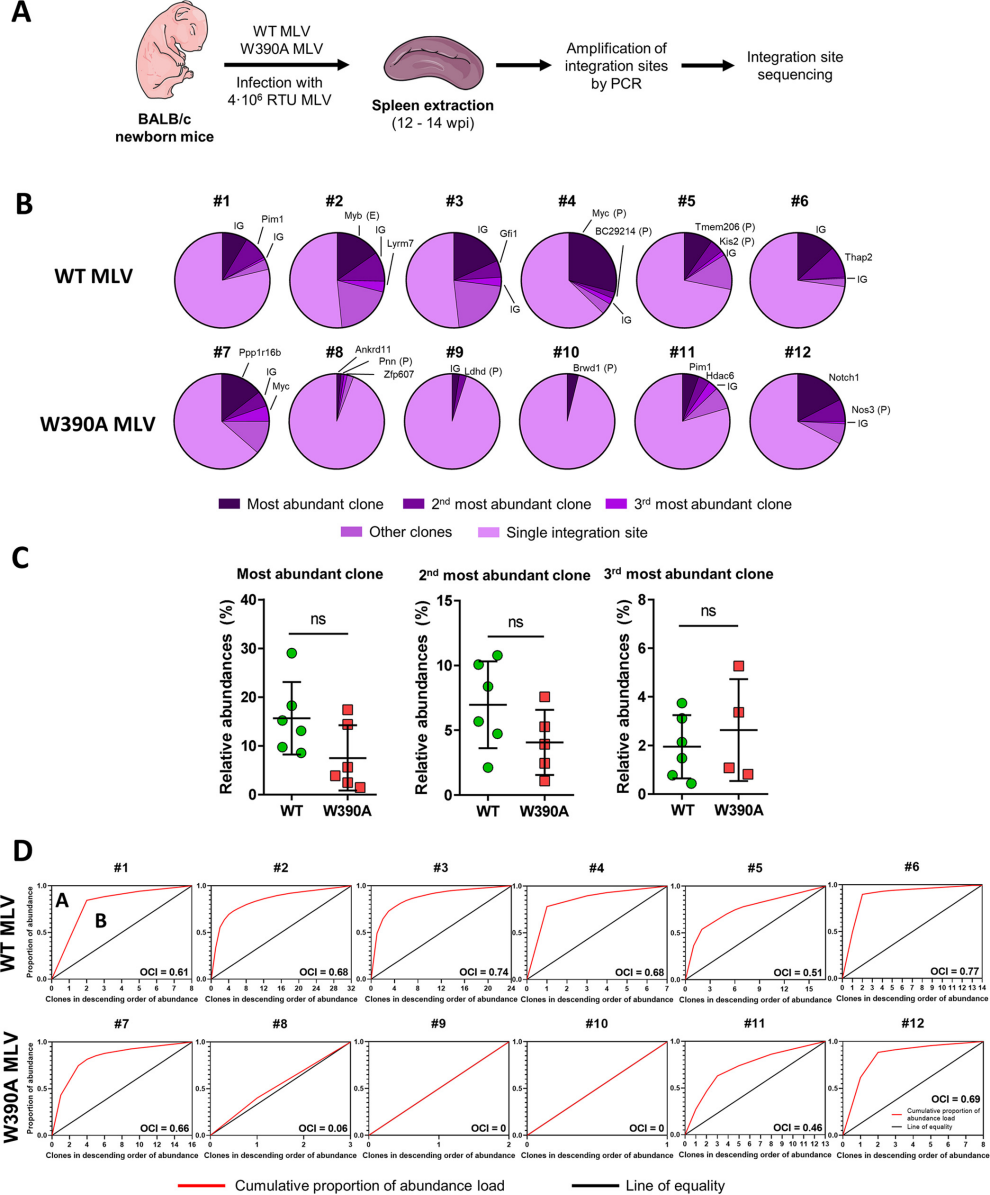
Clonal expansion of tumor cells in the spleen induced by WT MLV or W390A MLV. (A) Schematic representation of the workflow to determine the integration site selection of WT MLV and MLV W90A in mouse tumor cells at 12 to 14 weeks postinfection. (B) Pie charts representing the relative abundances of clones and single integration sites. Each pie chart corresponds to a single mouse infected with WT MLV or W390 MLV. Intergenic site (IG), gene, enhancer (E), or promoter (P) names indicate the MLV integration sites of the three most dominant clones in each mouse. (C) Relative abundances of the top three integration sites in mice infected with WT MLV or W390A MLV. No statistically significant difference was found using a Mann-Whitney test. (D) Cumulative fraction of MLV clones from WT MLV- or W390A MLV-infected mice. The oligoclonality index (OCI) was calculated as OCI = *A*/(*A* + *B*).

To assess if integration site selection was altered by the W390A mutation, six mice were infected with either WT MLV or W390A MLV and sacrificed 12 to 14 weeks after infection. In contrast to the above-described integration site analysis, at this time point, the mice had already developed lymphoma due to insertional oncogenesis. The integration profiles of both viruses were analyzed as described in the legend of Fig. 5A. Briefly, spleen cells from infected mice were isolated, and genomic DNA was extracted. Integration sites were determined by ligation-mediated PCR (LM-PCR), followed by high-throughput Illumina sequencing. A summary of the integration sites recovered by this analysis is provided in Table S4. An integration site detected ≥2 times is indicative of an MLV-infected parental cell that proliferates and generates a daughter cell with the MLV genome integrated into the same position of its genome (59). We refer to these groups of cells as “clones.” Fig. 5B displays the relative abundances of the clones. Mice infected with WT MLV presented one dominant clone that represented 10 to 30% of the total identified integration sites detected in our analysis. Overall, clones represented 20 to 50% of the integration sites detected. Other integration sites corresponded to single hits. In the case of W390A MLV, half of the mice showed a different pattern, with only a few clones being present (mice 8, 9, and 10) (Fig. 5B), while the other mice displayed a clonal pattern that resembled that of mice infected with WT MLV (mice 7, 11, and 12). No statistically significant difference was identified among the relative abundances of the three most dominant clones in mice infected with WT MLV or W390A MLV (Fig. 5C). Analysis of mice using the Bangham oligoclonality index (OCI) (60) revealed that clones generated in mice infected with WT MLV were associated with a more oligoclonal profile than clones generated in mice infected with W390A MLV, where three mice depicted a monoclonal profile defined by OCI values closer or equal to 0 (mice 8, 9, and 10). The distributions of clonal abundance statistically differed between clones generated by WT MLV and W390A MLV integration (Fig. S6A). WT MLV integration into intergenic positions generated three groups of clones in the tumors: low abundance (values of 2 to 3), medium abundance (values of between 4 and 6), and high abundance (values higher than 10) (Fig. S6B). In contrast, mutant virus infection generated a high number of clones with a low abundance together and few clones with a high abundance, regardless of integration into intergenic positions, gene bodies, or promoters. These results indicate that W390A MLV still induces the clonal proliferation of lymphoma cells.

**FIG 6:**
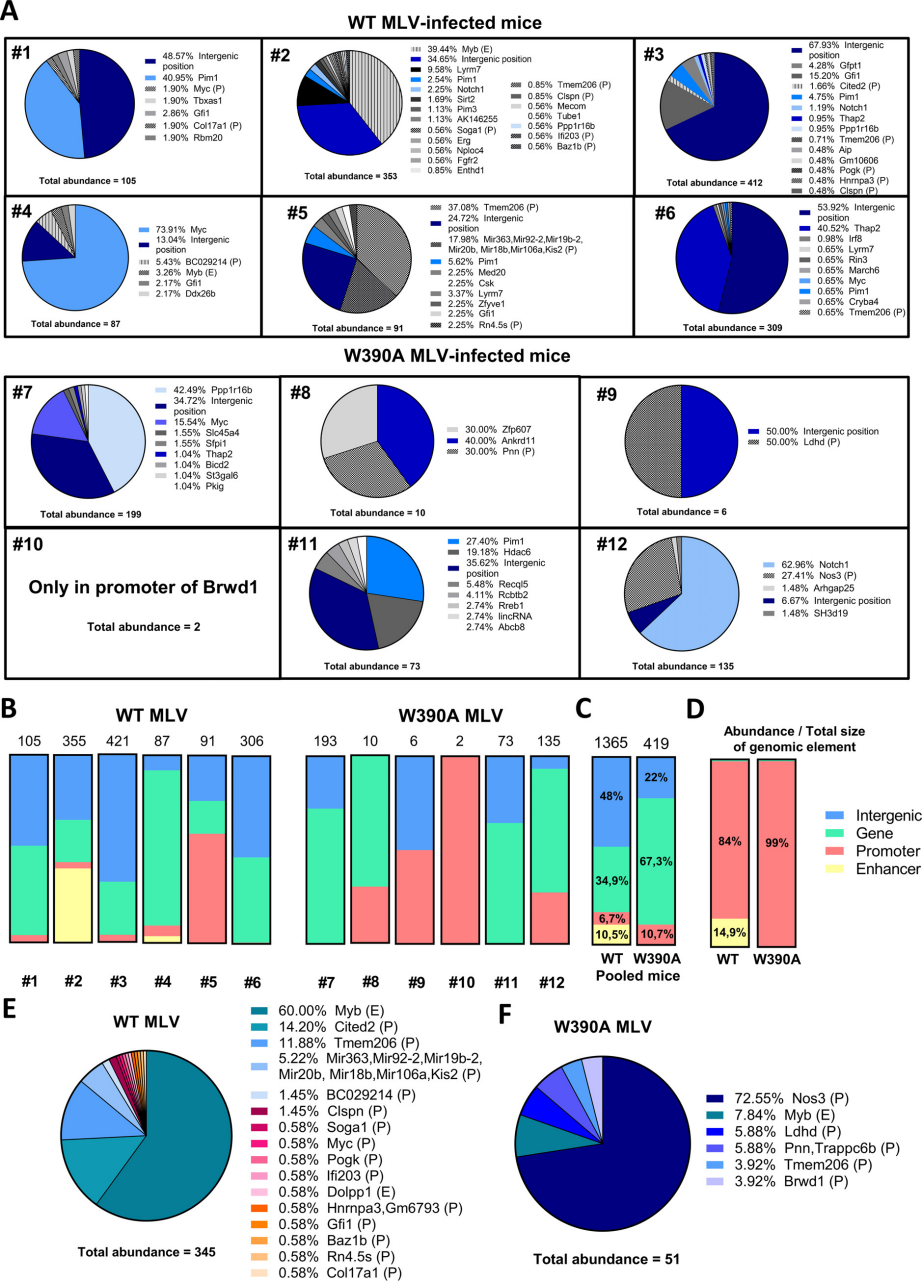
Distribution of MLV abundances in clones generated by WT MLV or W390A MLV insertion. (A) Pie charts depicting individual gene insertional profiles of splenic cells with clonal expansion from mice infected with 4 × 10^6^ RTU of either WT MLV or W390A MLV. The total abundance determined by NGS analysis is shown below each pie chart for both MLV constructs. The percentage before each gene name indicates the relative abundance of that gene. Genes with insertions of both WT MLV and W390A MLV are colored on a blue scale. (B) MLV abundances in intergenic positions (blue), gene bodies (green), promoters (red), and enhancers (yellow) in WT MLV- and W390A MLV-infected mice. The numeric mouse identifier is indicated below each bar. The total abundance of clonal integration sites in each mouse is indicated on top of the bar. (C) Pooled MLV abundances from intergenic positions (blue), gene bodies (green), promoters (red), and enhancers (yellow) in WT MLV (*n* = 6)- and W390A MLV (*n* = 6)-infected mice. The total abundance of pooled clonal integration sites from mice infected with either WT MLV or W390A MLV is indicated on top of the bar. The distribution of integration sites between WT MLV and W390A MLV was analyzed using a chi-squared test. The test reported a statistically significant difference between both distributions (*P* ≤ 0.0001). (D) MLV abundances from intergenic positions (blue), gene bodies (green), promoters (red), and enhancers (yellow) normalized by the total genomic size of each genomic feature. Data were normalized by dividing the percent integration site preference for each genomic feature by its own estimated total genomic size: intergenic positions (58.97%), genes (40.54%), promoters (0.05%), and enhancers (0.44%). Data represent results for six mice infected with WT MLV or W390A MLV. (E and F) Abundances in enhancer (E) or promoter (P) elements in spleen cells from mice infected with 4 × 10^6^ RTU of either WT MLV (*n* = 6) or W390A MLV (*n* = 6) isolated at 12 to 14 weeks postinfection. The total abundances of integration at enhancer and promoter sites are shown below each pie chart for both MLV constructs. The percentage before each gene name indicates the relative abundance of that gene. Integration into an enhancer or promoter element is indicated by E or P, respectively.

Next, we investigated the integration sites with a clonal origin in more depth. From the chromosome positions provided in Table S4, we identified genes targeted by MLV integration using an alignment to the NCBI37/mm9 mouse genome. WT MLV integration is characterized by a higher number of clonal integration sites (1,365 clones for WT MLV versus 284 clones for W390A MLV), (Fig. 6A). Both WT MLV and W390A MLV proviruses integrated into the *Myc*, *Notch1*, *Pim1*, *Ppp1r16b*, and *Thap2* oncogenes. In comparison with WT MLV, W390A MLV integrated less into intergenic positions but more frequently into the gene bodies of *Notch1* and *Ppp1r16b*. The integration of WT MLV and W390A MLV into these oncogene bodies had higher levels of clonal expansion and, therefore, abundances than those for other oncogene bodies found in this study. A chromosomal analysis of the MLV integration profile revealed that WT MLV preferentially integrated into chromosomes 5, 7, 10, and 12, while W390A MLV preferred chromosomes 2, 15, 17, and X (Fig. S7A). However, neither WT nor W390A MLV integration into the host genome was driven by gene density (Fig. S7B). In summary, these results reveal that lymphoma cells induced by W390A MLV display a significantly different integration profile than that of lymphomas induced by WT MLV.

### W390A MLV is targeted away from enhancers *in vivo*.

We further analyzed if these integration sites corresponded to enhancer or promoter elements using EnhancerAtlas 2.0. Fig. 6B and C show MLV integration site preferences for gene bodies, intergenic positions, enhancers, and promoters. In comparison, WT MLV displayed a relatively high abundance for enhancers (as shown for individual mouse 2 and mouse 4) and intergenic positions (on pooled data, 10.5% and 48% of the total abundance, respectively), while the abundance of W390A MLV was higher in promoters (as shown in individual mice 9 and 10) or gene bodies (mice 7, 8, 11, and 12) (on average, 10.7% and 67.3% of the total abundance, respectively). Strikingly, integration into promoters and enhancers was particularly enriched considering their small size in the genome. Normalization to genomic size showed that WT MLV has an 84% abundance in promoters and a 14.95% integration preference for enhancers, whereas W390A MLV shows a 99% abundance in promoters and no integration events in enhancers (Fig. 6D) (Table 1). Next, we pooled all the abundance values for enhancers and promoters from mice infected with either WT MLV or W390A MLV in the same pie chart (Fig. 6E and F). WT MLV preferentially showed clonal expansion from integration into the *Myb* enhancer (60% of the total sites of integration into enhancer or promoter elements) and the *Cited2* (14.2%) and *Tmem206* (11.88%) promoters (Fig. 6A). Integration into the *Myb* enhancer and the *Tmem206* promoter was also detected in W390A MLV-infected cells but at lower frequencies (7.84% and 3.92%, respectively) (*P* < 0.0001 by a χ^2^ test comparing distributions).

**TABLE 1:** Enrichment of integrations in enhancers and promoters for WT MLV and W390A MLV

MLV dose and construct	% integration/genomic size	
	Promoters	Enhancers
High dose		
WT	83.99	14.95
W390A	99.05	0
Low dose		
WT	49.85	48.79
W390A	76.11	17.96

The analysis of the integration profiles from individual mice revealed that each mouse has a unique integration profile driving the development of lymphoma (Fig. S8). In the case of W390A MLV, mice 7, 11, and 12 had a dominant clone in the tumor with integrations into the *Ppp1r16b*, *Pim1*, and *Notch1* oncogenes, respectively. In the case of WT MLV, mouse 2 displayed dominant integration at the *Myb* enhancer, while mouse 5 showed integrations into the *Tmem206* promoter. The lymphoma developed by mouse 4 was strongly driven by integration into the *Myc* oncogene.

To determine the potential impact of the viral titer, we also analyzed the integration site selection of WT MLV and W390A MLV in two groups of three mice infected with a lower dose (5 × 10^5^ RTU) (Fig. S9A). The results corroborate the findings in mice infected with a higher dose. Mice infected with W390A MLV at a lower dose displayed lower abundances for enhancers and promoters (Fig. S9B). When pooling the data from three mice, 3.1% of the total abundance was in enhancers, while 26.7% of the total abundance was found in promoters from WT MLV-infected mice (Fig. S9C). In contrast, mice infected with W390A MLV displayed only 1.3% of the total abundance in promoters and 2.7% in enhancers (Fig. S9C). Similar to mice infected with a higher dose of MLV, integration and clonal expansion from enhancers and promoters were enriched (for WT MLV, 49.9% in promoters and 48.9% in enhancers; for W390A MLV, 76.1% in promoters and 18% in enhancers) (Fig. S9D) (Table 1). All mice displayed a polyclonal integration profile, except for mouse 29, which displayed an oligoclonal integration profile (Fig. S9E). Integration into the *Myb* enhancer was found in WT MLV-infected mice (mice 29 and 30). In mice infected with W390A MLV, integration into *Notch1* and *Pvt1* oncogene bodies was evident (Fig. S9F).

Finally, we analyzed if MLV insertional mutagenesis resulted in truncated oncogenes using next-generation sequencing (NGS) analysis of WT MLV- or W390A MLV-infected mice. Integrations of both WT MLV and W390A MLV into *Thap2*, *Pim1*, and *Notch1* occurred toward the 3′ end of the gene body (Fig. S10A). However, both WT and W390A MLV integrated into the central part of the *Ppp1r16b* gene. The integration preferences of WT MLV and W390A MLV differed with respect to the *Myc* gene; whereas WT MLV integrated near the 3′ end, W390A MLV integrated near the 5′ end. Previous studies on the mechanism of insertional mutagenesis reported that MLV insertion near gene bodies can increase gene expression due to intrinsic enhancer and promoter elements within the long terminal repeats (LTRs) of MLV (6, 61). Common targets for MLV integration are regions near the *Lmo2*, *Mecom2*, and *Prdm16* genes in humans (62) or mice (61). An intragenic insertion into these genes was not identified in our integration site analysis, but we analyzed the expression of these genes using RT-qPCR in spleen cells from a different set of MLV-infected mice, isolated at 12 to 14 weeks postinfection, by qPCR. The results show that MLV 63.2, WT MLV, and W390A MLV effectively increase the expression of *Mecom2* and *Prdm16* (Fig. S10B). However, 63.2 MLV and WT MLV were able to induce a stronger increase in *Mecom2* and *Prdm16* expression than W390A MLV.

Although most WT MLV integrations took place at intergenic positions and gene bodies, there was a relative enrichment for enhancers and promoters that represent only 0.49% of the mouse genome. The proportions of integration into enhancers and promoters in mice infected with WT MLV were 17.2% with the high dose and 29.8% with the low dose. BET-independent W390A MLV IN integrated away from enhancers in tumorigenic cells. At a high dose, only 10.7% of W390A MLV integrated into enhancers/promoters, and with a low dose, only 4% integrated into enhancers/promoters (Table 1).

## DISCUSSION

### BET proteins are not required for MLV *in vivo* integration and replication.

**FIG 7:**
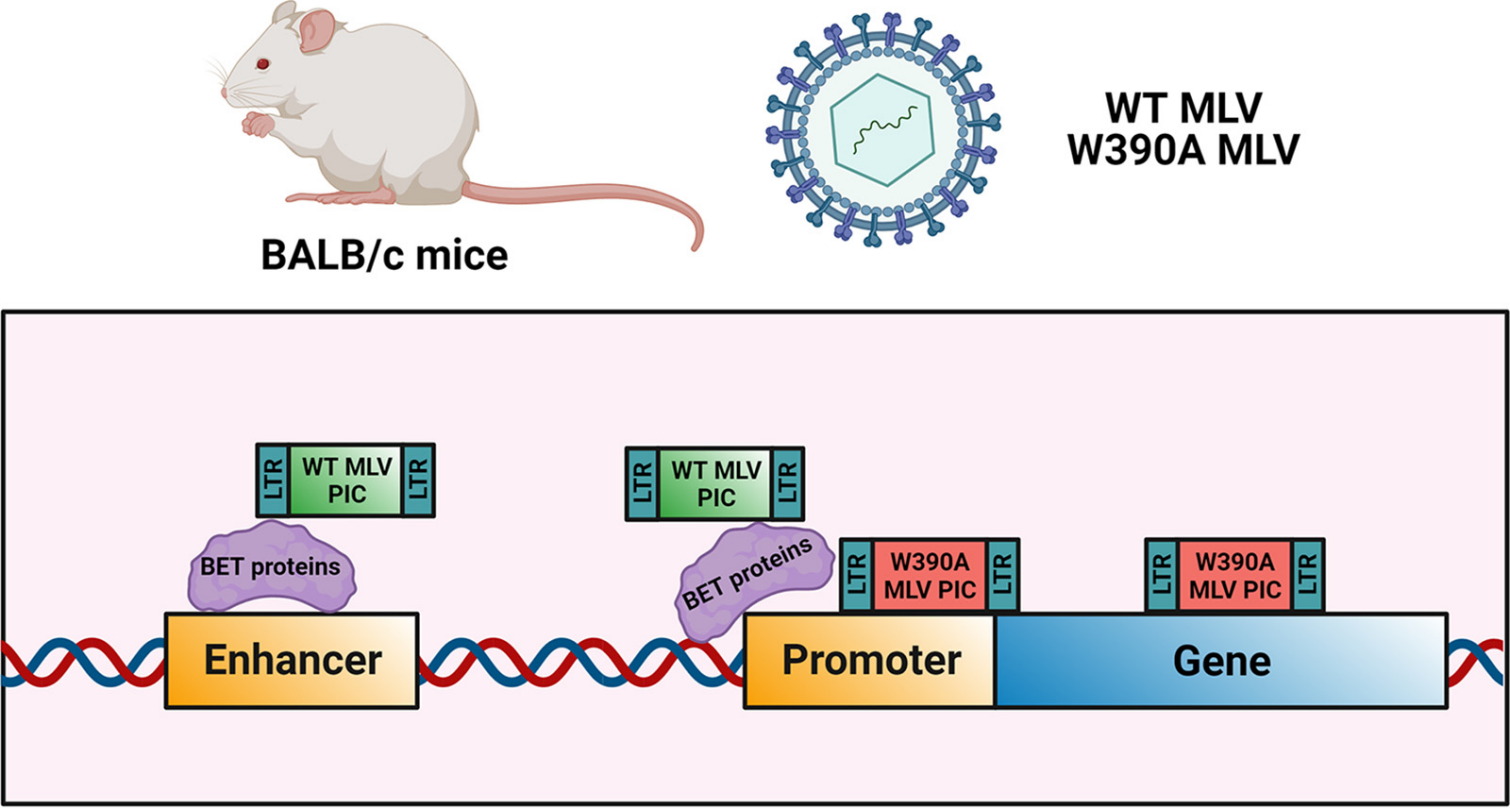
Model of MLV integration *in vivo*. The MLV preintegration complex (PIC) is targeted to enhancers and promoters by the BET proteins, while the lack of an interaction between MLV PIC and BET proteins retargets integration toward promoters and gene bodies by the implication of an unknown host factor or chromatin feature or a direct implication of the MLV integrase. The strong MLV LTR enhancer/promoter can drive oncogenesis, even after retargeting.

Our work unequivocally demonstrates that the interaction between BET proteins and MLV IN is not essential for MLV integration, replication, and lymphomagenesis *in vivo*. We reported previously that the W390A mutation abrogates the total interaction with BET proteins using an AlphaScreen assay, to a level similar to that with the MLV IN_1–381_ construct that lacks the C-terminal tail of the MLV IN (19). In the present study, we demonstrate that W390A MLV can replicate under *in vivo* conditions, which strongly suggests that additional host or viral factors may be involved during integration (Fig. 7). We have shown that the *in vivo* integration of W390A was retargeted away from TSSs and CpG islands but to a lesser extent than with *in vitro* data (27, 28). This difference might be explained by the implication of different host cofactors considering that the cell types analyzed in the present study (splenic cells) and those analyzed in previous studies (CD34^+^ hematopoietic cells, SupT1 cells, and HeLa cells, among others) are not the same. While the potential alternative cofactor remains unknown, these findings are in line with our previous observations for HIV integration. We and others reported previously that heparing binding growth factor (HDGF)-related protein 2 (HRP2) can replace LEDGF/p75 in LEDGF/p75 knockout cells (63, 64).

Our data show that the BET-independent MLV W390A mutant integrates and replicates in mice to an extent similar to that of the WT virus. This suggests that MLV IN either directly interacts with chromatin or uses alternative host factors or mechanisms. A likely candidate is p12, a cleavage product of MLV Gag that has been implicated in the early steps of the replication cycle of MLV (65, 66) as well as virion production (67). The MLV protein p12 can tether the MLV preintegration complex to host chromosomes (68, 69) by the phosphorylation of serine 61, the key event to tether the MLV preintegration complex to chromatin (49, 70). Yueh and Goff confirmed that the lack of p12 phosphorylation of the serine amino acids at positions 61 and 78 results in the impaired formation of LTR circles (71), but these mutants could still release normal levels of mature virions, suggesting that p12 may not be essential for the replication of MLV. On a similar trend, another study showed that alteration of p12-mediated tethering had no effect on MLV integration into TSSs (72). The relative roles of viral p12 and cellular BET proteins in tethering and targeting integration await further investigation. Possibly, tethering by p12 of MLV PICs to mitotic chromosomes is an essential feature while BET-mediated targeting is an optional feature that still may have evolutionary advantages since BET proteins remain bound to mitotic chromosomes and are associated with early gene transcription after the completion of mitosis (73).

### The MLV-BET interaction is not the essential driver of tumorigenesis.

BALB/c mice infected with either WT MLV or W390A MLV developed the same disease phenotype. Upon evaluation, we identified an enlargement of the thymus and spleen, both typical of lymphoma (74). Histopathology identified the same type of malignant population in both WT MLV- and W390A MLV-infected mice by H&E staining. These malignant cells are transcriptionally and mitotically more active than cells from mice injected with PBS. MPO and PAX-5 staining revealed the lack of B cells, granulocytes, and monocytes in spleen lymphomas. Histopathology and marker analyses revealed strong CD3 staining in mice infected with WT or W390A MLV, in contrast to mice injected with PBS, indicating that these mice developed lymphomas of T-cell precursors (75). Moreover, the lymphomas were characterized by polyclonal expansion. Lymphomas induced by retroviruses often display a polyclonal phenotype (76) due to integration into multiple loci in the same cell, which eventually leads to oncogenesis by the disruption of gene networks that control proliferation (77). In summary, all tumors induced by WT MLV infection were characterized by a polyclonal profile. On the other hand, tumors induced by W390A MLV infection displayed a mixed profile consisting of polyclonal, oligoclonal, and monoclonal tumors (in the latter case for 1 out of 6 mice, corresponding to mouse 10).

### The lack of BET-MLV IN interaction still yields lymphomas through insertional mutagenesis in oncogene bodies.

In the context of gene transcription, BRD4 plays an important role in superenhancers (SEs) (78). SEs have been defined as clusters of enhancers that are occupied by a high density of multiple transcription factors (79). In this sense, BRD4 and MED1 can act as coactivators of the SEs, and their presence defines an SE (80). In cancer cells, the acquired cancerous phenotype relies on abnormal transcription promoted by superenhancers (78). Although BRD4 may acquire this function at later stages when a lymphoma develops in MLV-infected mice, the tethering mechanism whereby MLV IN binds to BRD4 may point to a role during the retroviral integration step. We show that MLV preferentially integrates into promoters and enhancers under *in vivo* conditions, which is in line with results reported previously by other groups using cell lines and primary cells (5, 6, 81). For WT MLV, integration into enhancers was strongly enriched, while W390A MLV integration was enriched in promoters and gene bodies. Normalization of the relative abundance in Fig. 6C to genomic size resulted in an integration preference of >98% (WT MLV, 98.9%; W390A MLV, 99%). Whereas WT MLV preferentially integrates into promoters and enhancers, BET-independent MLV preferentially integrates into promoters and gene bodies. The role of BDR4 is to target integration into enhancers known to interact with BRD4. W390A MLV integration into oncogene bodies has major implications for the development of lymphomas in infected mice. As shown in Fig. 6A, 4 out of 6 mice infected with W390A MLV had a dominant clone corresponding to integration into an oncogene body. Opposite this, only 1 out of 6 mice infected with WT MLV had a dominant clone as a result of integration into an oncogene body. Therefore, not only are integrations into gene bodies more frequent (as shown in Fig. S8 in the supplemental material), but also, once W390A MLV integrates into oncogene bodies, these genes are selected for driving the development of lymphomas in W390A MLV-infected mice. Still, W390A MLV targets a smaller set of oncogenes. Perhaps this is due to less efficient BET-independent integration or the use of a redundant targeting mechanism with another (host) factor.

WT MLV integration into the enhancer of *Myb* displayed strong clonal expansion (82), while W390A MLV was retargeted away from *Myb*, a common target for MLV integration. Also, WT MLV integrated into *Tmem206* and *Cited2* promoters, which induced strong clonal expansion. Both *Tmem206* and *Cited2* were recently described as oncogenes involved in lymphoma (83, 84) in human cells. Overexpression of *Myb* has been detected in both acute myeloid leukemia and non-Hodgkin lymphoma (85, 86), while *Tmem206* and *Cited2* overexpression was detected in colon cancer and prostate cancer, respectively. The molecular mechanism implicated in oncogenesis driven by *Tmem206* is the activation of the AKT signaling pathway (87). Moreover, both parental 63.2 MLV and WT MLV enhanced the expression of *Mecom2* and *Prdm16* (Fig. S10), while the increase in expression induced by W390A MLV was 2-fold lower, compatible with a lower integration frequency of W390A MLV near enhancers and/or promoter elements. The W390A mutation in MLV IN significantly retargeted viral integration away from enhancers toward oncogenes bodies such as *Notch1* and *Ppp1r16b*. *Notch1* has been widely described as one of the driver genes in T-cell acute lymphoblastic leukemia/lymphoma (T-ALL) (88–90), while *Ppp1r16b* can promote aggressive lymphomas in mice (91). The truncation of *Notch1* can generate lymphomas by the loss of the PEST domain located in the 3′ end of the *Notch1* gene, which leads to an enhanced protein half-life and, therefore, longer activity as a transcription factor in the nucleus (92). Insertional mutagenesis of *Notch1* can also result in deletions in the 5′ end that activate intragenic promoters driving the expression of truncated transcripts that lack the negative regulatory region (NRR) (93). In the case of *Ppp1r16b*, a previous study reported a modest increase in the expression of *Ppp1r16b* in several MLV-induced lymphomas (91). Such an increase was more relevant when an MLV insertion happened at the *Ppp1r16b* locus.

As such, by combining the data for all the mice used in this study infected with a higher dose of MLV (6 mice for WT MLV and W390A MLV) (Fig. 5 and 6) and a lower dose of MLV (3 mice for WT MLV and W390A MLV) (Fig. S9), our results indicate that tumors induced by WT MLV (44.4%, or 4 out 9 mice, corresponding to mice 2, 5, 27, and 28) could be caused by the overexpression of oncogenes (such as *Myb*) resulting from integration into an enhancer or promoter near such an oncogene, while the tumors induced by W390A MLV (77.7%, or 7 out of 9, corresponding to mice 7, 8, 11, 12, and 30 to 32) could be generated frequently after insertional mutagenesis into an oncogene body.

### Importance of the C-terminal tail of MLV IN in viral replication.

The C-terminal deletion of MLV IN has a major impact on the replication of MLV since ΔC MLV was associated with reduced viral replication in comparison with W390A MLV. Although the reason for this is unknown at the moment, we speculate that MLV IN might have an effect on the assembly and release of newly formed MLV particles. The role of the MLV IN-BET protein interaction in an *in vivo* context was previously studied by Loyola et al. (51). In contrast to our present study with the W390A mutant, distinct BET-independent MLV constructs were used. First, a construct carrying a truncation of 23 amino acids from the C-terminal tail of MLV IN (MLV IN XN) (94) deficient for BRD4 interactions was generated. Next, an MLV IN construct lacking the terminal peptide (TP^−^) was made, including stop codons in the non-*env* reading frames to prevent the restoration of WT IN. The *MYC/Runx2* tumorigenesis mouse model was used by Loyola et al. (51), which is characterized by the spontaneous development of lymphomas (95). As such, it does not reflect primary insertional lymphomagenesis upon MLV infection. We used healthy BALB/c mice instead, which develop tumors only upon MLV infection. Both the MLV IN XN and MLV IN TP^−^ constructs showed a 10-fold decrease in *in vitro* infectivity, which corresponds more or less to the 25-fold decrease in infectivity seen with ΔC p63.2 MLV, lacking 27 C-terminal amino acids, compared to WT MLV and W390A MLV 24 h after infection of NIH 3T3 cells (Fig. S1D). While W390A MLV replicated to WT levels in our mouse model, no evaluation of infectivity under *in vivo* conditions was performed by Loyola and colleagues (51), but it can be deduced that the replication of MLV with IN C-terminal deletions was hampered.

Whereas W390A MLV-induced lymphomagenesis showed a trend toward reduced mouse survival, the TP-deleted MLV clones described previously by Loyola and colleagues resulted in the increased survival of *MYC*/*Runx2* mice in 15 days (51). This result may, however, reflect reduced virus replication *in vivo*. In the latter study, recombination with endogenous retrovirus *Pmv20* was able to revert the expression of the terminal peptide, confounding the analysis (51). We used BALB/c mice, characterized for the presence of polytropic and xenotropic endogenous retroviruses (96). Although mice infected with W390A MLV displayed a higher number of single mutations in MLV IN, suggesting selective pressure, no revertants due to mutagenesis or recombination were observed (Fig. S2). As for the integration site analysis, both studies report less integration into CpG islands and TSSs with BET-independent virus. In conclusion, both studies report BET-independent MLV integration in mice. In both studies, BET-independent MLV is retargeted but still associated with lymphoma. In the previous study, a poor replication capacity and recombination hampered the analysis of the role of BET interactions in lymphomagenesis, while our setup allowed a more detailed pathological and genomic analysis.

### Genotoxicity of BET-independent MLV vectors.

One important application of MLV integration studies is the development of safer MLV-based vectors for gene therapy. Previously, it was shown that the safety of SIN MLV vectors varies in different tissues (64). In this study, we have used the W390A mutation in MLV IN to obtain a better understanding of the relevance of BET proteins in the development of lymphoma. Overall, our results indicate that the prevention of the MLV IN-BET interaction was sufficient to retarget integration away from enhancers but not enough to prevent the development of lymphomas by insertional mutagenesis. Our analysis points to the additional role of the enhancer/promoter in the MLV LTR in driving oncogenesis. Second-generation SIN MLV vectors take this feature into account and are associated with an increased safety profile (48). An *in vivo* analysis of BET-independent SIN MLV vectors is warranted. Such MLV vectors may have a better genotoxic safety profile.

### Evolutionary advantage of the use of BET proteins for MLV integration.

If the interaction of MLV IN with BET is dispensable for MLV replication *in vivo* and lymphomagenesis, why did MLV evolve this mechanism? Although different explanations can be thought of, BET-mediated integration may provide a more efficient pathway. First, in the present study and the study reported previously by Loyola et al. (51), the number of integrations detected in mice infected with either W390A MLV or tail peptide-deleted MLV was lower than the number of integrations for WT MLV. In this sense, BET-dependent MLV may be able to establish a higher number of proviruses inside the host genome and a greater chance for productive infection. Second, we show how BET proteins direct MLV integration *in vivo* toward enhancers and promoters. Integration at these sites ensures high transcriptional activity. In fact, BRD4 can remain bound to several loci to remember the gene transcriptional profile after mitosis (73), a process that MLV relies on for completing nuclear entry. Integration at these sites may guarantee that the transcriptional machinery can initiate the transcription of the MLV genome and continue the replication cycle. The strong enhancer/promoter of the MLV LTR clearly contributes to the high transcriptional activity, which, in contrast to HIV, is independent of a transactivator such as Tat. Lymphomagenesis may not be an intrinsic feature of the MLV replication cycle but may be a bystander effect. Although this is not beneficial for a pathogen, in the case of mice, horizontal transmission to offspring is guaranteed before their death considering how early in their life span mice reach adulthood. The integration preference near enhancers and promoters and the strong LTR promoter all contribute to the risk for lymphomagenesis. Whether integration near oncogenes is stimulated by BRD4 interactions or whether BRD4-mediated integration near oncogenes is selected for clonal proliferation is not fully clear. Our data point toward the second scenario, but more detailed analyses are required. The multipronged mechanism to ensure proviral expression after transcription and, thus, the relative absence of latency may be associated with an intrinsic risk for insertional mutagenesis, clonal expansion, and tumorigenesis.

## MATERIALS AND METHODS

### Cell culture.

The human kidney HEK 293T/clone 17 (293T/17) cell line was acquired from the ATCC (ATCC CRL-11268). The NIH 3T3 cell line was a kind gift from Johan Van Lint from the Laboratory for Protein Phosphorylation and Proteomics (KU Leuven). Both cell lines were maintained in Dulbecco’s modified Eagle’s medium (DMEM)-GlutaMAX I (Gibco, Thermo Fisher Scientific) with 10% fetal bovine serum (FBS) (Gibco) at 37°C with 5% CO_2_.

### Animal tests.

BALB/c mice were acquired from Janvier Labs (Le Genest-Saint-Isle, France). All the experiments and procedures with animals were done according to European Directive 2010/63/EU for the protection of animals used for scientific purposes. The procedures were also reviewed by the Ethical Commission of Animal Tests (Ethische Commissie Dierproeven) of KU Leuven (internal approval number P210/2014, extended P116/2019). An overview of mice used in this study (including a reference to each figure and experiment) is shown in Table S1 in the supplemental material.

### MLV constructs.

Mutations in MLV IN of pNCS (71) were done using the site-directed ligase-independent mutagenesis (SLIM) cloning technique (97). Next, the SgrAI/SalI fragment was shuttled into the MLV molecular clone p63.2, which was obtained from Susan R. Ross (98). Plasmid p63.2 is a pBR322 plasmid containing the wild-type Moloney MLV provirus clone (99, 100). The C-terminal integrase sequences from the generated MLV clones are shown in Fig. S1.

### MLV production.

Approximately 5.5 × 10^6^ HEK 293T cells were plated into a petri dish. For *ex vivo* experiments, DMEM-GlutaMAX I (Gibco, Thermo Fisher Scientific) with 10% FBS (Gibco) and 50 μg/mL gentamicin (Gibco) was used. For *in vivo* experiments, Opti-MEM I-GlutaMAX I (Gibco) supplemented with 2% FBS and 50 μg/mL gentamicin (Gibco) was used. Twenty-five micrograms of the viral plasmid and 100 μL of branched polyethyleneimine (PEI) (pH 7.4) (Sigma-Aldrich) were added to the cell culture. At 24 h posttransfection, fresh medium was added. Medium was harvested at 48 and 72 h posttransfection and filtered through a 0.45-μm filter (Merck Millipore, Overijse, Belgium). MLV was concentrated by ultrafiltration using Vivaspin 50-kDa-molecular-weight-cutoff (MWCO) filter tubes (Merck Millipore).

### Semiquantitative PCR and RT-qPCR.

RNA extractions were done using the Aurum total RNA kit (Bio-Rad) according to the manufacturer’s instructions. RNA was quantified using an SP062 nanophotometer (Implen, Munich, Germany), and 5 μg of each sample was taken for reverse transcription. RT-PCR was done using the high-capacity cDNA reverse transcription kit (Applied Biosystems, Thermo Fisher Scientific, Brussels, Belgium).

Semiquantitative PCR was performed using iProof high-fidelity (HF) kit polymerase (Bio-Rad, Temse, Belgium) according to the manufacturer’s instructions. PCR was done on the T personal Biometra thermocycler (Westburg). The cycling conditions were 98°C for 45 s and 40 cycles at 98°C for 20 s, 55°C for 20 s, and 72°C for 90 s, ending with 1 cycle at 72°C for 5 min. Semiquantitative PCR products were analyzed by gel electrophoresis with a 1% agarose gel (catalog number 15510-027; Invitrogen), using the Bio-Rad PowerPac basic electrophoresis power supply at 140 V for 40 min. A 1-kb GeneRuler (Thermo Fisher Scientific) was used as a ladder.

Quantitative PCR (qPCR) was performed using LightCycler 480 SYBR green I master mix (reference number 04707516001; Roche) or IQ Supermix (reference number 1708860; Bio-Rad) for the glyceraldehyde-3-phosphate dehydrogenase (GAPDH) gene with a 6-carboxyfluorescein (FAM)–6-carboxytetramethylrhodamine (TAMRA)-labeled probe. Primer concentrations were adjusted to a final concentration of 300 nM. Two-step qPCR was done using the LightCycler 480 instrument (Roche, Anderlecht, Belgium). The activation cycle was performed at 95°C for 10 min, followed by 40 cycles at 95°C for 10 s and then 60°C for 30 s. Cooling was done by 1 cycle at 37°C during 1 s. Primer sequences used for SYBR green product-enhanced reverse transcriptase (SG-PERT) assays, quantitative PCR, semiquantitative PCR, and the amplification of integration sites are listed in Tables 2 and 3.

**TABLE 2:** Primers used for SG-PERT, quantitative PCR, and semiquantitative PCR

Gene	Sequence			Technique
	Forward primer	Reverse primer	Probe	
MS2 phage PERT	TCCTGCTCAACTTCCTGTCGAG	CACAGGTCAAACCTCCTAGGAATG		SG-PERT
MLV Env	CCTACTACGAAGGGGTTG	CACATGGTACCTGTAGGGGC		qPCR
MLV IN	AAGCTCAGGCCAGGTAGAAAGAA	TCCCGATCTCCATTGGTTACCT		Revertant analysis
Lmo2	ACCGCTACTTCCTGAAAGCC	GACACCCACAGAGGTCACAG		qPCR
Mecom2	CGACAGAGGAGTGGGAGAAG	TCTAAGATCTTCCGATTTCTACGGC		qPCR
Prdm16	CAGCACGGTGAAGCCATTC	GCGTGCATCCGCTTGTG		qPCR
Rodent GAPDH	TGTGTCCGTCGTGGATCTGA	CCTGCTTCACCACCTTCTTGA	FAM-CCGCCTGGAGAAACCTGCCAAGTATG-TAMRA	qPCR

**TABLE 3:** Primers used for amplification of integration sites^*a*^

Amplification step	Sequence	
	Oligonucleotide 1	Oligonucleotide 2
PCR1	ATTCGAAGCGAACAACACTGAC	CCTCTTGCAGTTGCATCCGAC
PCR2	AATGATACGGCGACCACCGAGATCTACACCAGGACTGACGCTATGGTAATTGTTGACCCTTCGGATTACCCG	CAAGCAGAAGACGGCATACGAGATXXXXXXXXXXXXAGTCAGTCAGCCTGATTGACTACCCGTCAGCGG

a X corresponds to nucleotide positions used as barcodes for sequencing. The set 1 oligonucleotides from PCR1 and PCR2 bind to the linker, while the set 2 oligonucleotides bind to the MLV LTR.

### MLV titration by a SYBR green product-enhanced reverse transcriptase assay.

Titration was done as described previously (101). MLV was produced in NIH 3T3 cells. The supernatant was filtered through a 0.45-μm filter (Millipore). The MLV supernatants and standards were lysed using 2× lysis buffer (0.25% Triton X-100, 50 mM KCl, 100 mM Tris-HCl, 40% glycerol [pH 7.4]) with incubation at room temperature for 10 min. After lysis, 1× sample dilution buffer was added to each sample and standard [10× dilution buffer contains 50 mM (NH_4_)_2_SO_4_, 200 mM KCl, and 200 mM Tris-HCl (pH 8.3)] at a final dilution of 1:10. Standards were diluted in a dilution series of 1:5. PCR was performed with a final concentration of 0.25 U/μL Fermentas Truestart Hot Start *Taq* DNA polymerase (Thermo-Fisher) in 100 μL of a solution containing 2× reaction buffer [5 mM (NH_4_)_2_SO_4_, 20 mM KCl, 20 mM Tris-HCl (pH 8.3)], 10 mM MgCl_2_, 0.2 mg/mL bovine serum albumin (BSA), a 1:10,000 dilution of SYBR green I, 500 μM deoxynucleoside triphosphates (dNTPs), 4 μM forward primer MS2 phage PERT, 4 μM reverse primer MS2 phage PERT (both listed in Table 2), and 2 μg/mL MS2 RNA. The thermocycler used was the LightCycler 480 instrument (Roche). The cycling conditions were 37°C for 60 min, 95°C for 5 min, and 45 cycles at 95°C for 5 s, 55°C for 5 s, and 72°C for 15 s. The final cycle was done at 81°C for 10 s.

### Quantification of viral DNA in cells from the spleen, thymus, and blood.

Genomic DNA was extracted from mouse spleen, thymus, and blood samples using the commercial GenElute mammalian genomic DNA miniprep kit (Sigma-Aldrich). Two-step quantitative PCR was performed using MLV Env primers (102) (listed in Table 2) that amplify the region of the MLV genome located between bp 6546 and 6765 (NCBI reference sequence accession number NC_001501.1). Rodent GAPDH was used as a housekeeping gene using the LightCycler 480 instrument (Roche, Anderlecht, Belgium). The activation cycle was performed at 95°C for 10 min, followed by 40 cycles at 95°C for 10 s and then 60°C for 30 s. Cooling was done in 1 cycle at 37°C during 1 s.

### Quantification of MLV titers by coculture.

For the quantification of MLV titers by coculture, round, 12-mm-diameter BioCoat coverslips (Corning; Fisher Scientific, Merelbeke, Belgium) were first added to a 24-well plate (Nunc; Thermo Scientific, Asse, Belgium). Next, approximately 1.5 × 10^5^ NIH 3T3 cells were seeded into each well. After 24 h, NIH 3T3 cells were cocultured with 10^4^ cells from the spleen or thymus for 24 h. Next, NIH 3T3 cells were rinsed with PBS and fixed with 4% paraformaldehyde. For staining, a primary antibody against the MLV protein p12 was obtained from hybridoma alpha CA cells (ATCC CRL-1890). The primary antibody was incubated for 1 h. Goat anti-mouse biotin (Dako Denmark, Glostrup, Denmark) was used as a secondary antibody, and samples were incubated for 20 min, followed by an incubation step with streptavidin-horseradish peroxidase (HRP) (Dako Denmark) for 30 min. Next, 3,3’-diaminobenzidine (DAB) staining was done. Coverslips were mounted using Mowiol 4-88 (Calbiochem, Merck). Infected cells were counted with a Leica DMR microscope (×40 magnification).

### Mouse infection.

One-day-old mice were infected by intraperitoneal injection with 5 or 50 μL of 8·10^4^ RTU/μL of MLV, as indicated for each experiment. Moribund mice were sacrificed when the disease reached a late stage, around 12 weeks postinfection, by cervical dislocation. Before sacrifice, mice were weighed. Samples of whole blood, thymus, and spleen were obtained for histological analysis.

### Blood sampling and cell counting.

Blood samples were taken from the submandibular vein by puncture, and blood was stored in MAP microtubes coated with 1 mg EDTA (Becton, Dickinson, Franklin Lakes, NJ). The blood was diluted 1:10 in PBS, and blood cell counting was performed using the Siemens (Munich, Germany) Advia 2120 hematology analyzer. For further confirmation, blood smears were performed, and blood cells were identified and counted microscopically to corroborate the results from the automatic cell counter.

### Histological sections.

Tissues were fixed in 10% neutral buffered formalin for 24 h and embedded in paraffin. Formalin-fixed and paraffin-embedded sections were cut at a 3-μm thickness. Automated hematoxylin and eosin (H&E) staining was performed on a Leica (Wetzlar, Germany) ST5010 Autostainer XL instrument for all samples.

### Integration site sequencing.

Integration site sequencing was done as described previously (103). Briefly, mice were injected with WT MLV or W390A MLV. At 3 and 5 weeks postinfection or at a later stage of the disease (12 to 14 weeks postinfection), mice were sacrificed, and spleens were collected. Genomic DNA was extracted using the GenElute mammalian genomic DNA miniprep kit (Sigma). Sonication with the Covaris M220 instrument was used to shear genomic DNA randomly, and DNA linkers were ligated to the sheared DNA. Integration sites were amplified by nested PCR using iProof high-fidelity kit polymerase (Bio-Rad). Two sets of oligonucleotides that bind to the MLV LTR and the linker were used for PCR (103) (sequences are listed in Table 3), whereby Illumina sequencing adapters were linked to the second oligonucleotide. PCR products were purified using AMPure XP magnetic beads and sequenced using Illumina MiSeq (Illumina Inc., San Diego, CA, USA) paired-end reads with 300 cycles by the Leuven Genomics Core (KU Leuven). The relative abundance of each unique integration site was calculated according to the following formula: relative abundance = (abundance of integration sites/total abundance) × 100.

### Revertant analysis.

Genomic DNA was extracted using the GenElute mammalian genomic DNA miniprep kit (Sigma). PCR using iProof polymerase was performed with the following reaction mix: 10 μL iProof HF buffer, 1 μL dNTP mix (250 μM each), 1 μL forward primer (5 μM), 1 μL reverse primer (5 μM), 1 μL DNA template (20 ng), 35.5 μL nuclease-free water, and 0.5 μL iProof polymerase. The primers used for PCR (both listed in Table 3) amplify the region of the MLV genome located between bp 4988 and 5658 (NCBI reference sequence accession number NC_001501.1). The cycling conditions were 98°C for 30 s, followed by 30 cycles at 98°C for 10 s, 54°C for 20 s, and 72°C for 30 s. The final cycle was done at 72°C for 10 min. The PCR product was sequenced by LGC Biosearch Technologies by Sanger sequencing.

### Statistics and software.

*P* values associated with each graph are represented by asterisks in the figures (*, *P* ≤ 0.05; **, *P* ≤ 0.01; ***, *P* ≤ 0.001; ****, *P* ≤ 0.0001). The statistical tests used to analyze significant differences between conditions are indicated in each figure legend. INSPIIRED software was used to analyze the integration sites (103). Integration site positions were aligned to the NCBI37/mm9 mouse genome to identify insertions in mouse genes using USCS Genome Browser. The genomic sizes of enhancers in mouse spleens were obtained from EnhancerAtlas 2.0. The genomic sizes of genes were obtained from the UCSC gene database (NCBI37/mm9, July 2007 assembly). The genomic sizes of mouse promoters were obtained from the EPD new promoter database (GRC38/mm10, December 2011 assembly). EnhancerAtlas 2.0 was used to analyze integration into enhancer and promoter features (104). GraphPad Prism 8 was used to make graphs and statistical calculations.
