# Supplementary material for: BET-Independent Murine Leukemia Virus Integration Is Retargeted In Vivo and Selects Distinct Genomic Elements for Lymphomagenesis
Source: Microbiol Spectr. 2022 Jul 19;10(4):e01478-22. doi: 10.1128/spectrum.01478-22 (PMC9431007; doi:10.1128/spectrum.01478-22)
Supplement: Supplemental file 5 — Supplemental material. Download spectrum.01478-22-s0005.pdf, PDF file, 5.7 MB [file spectrum.01478-22-s0005.pdf]

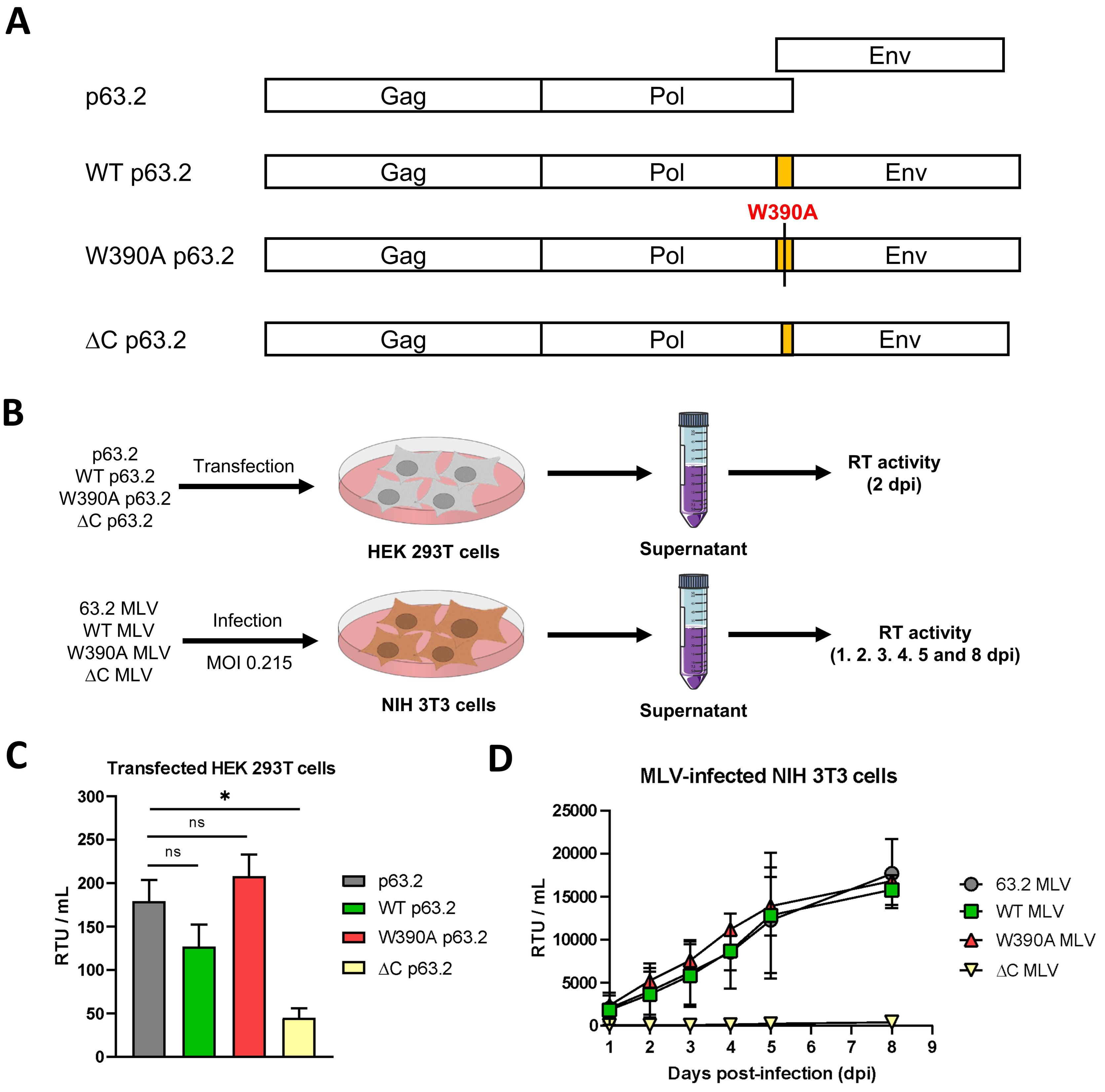

A
Env
p63.2
Gag
Pol
WT p63.2
Gag
Pol
Env
W390A
W390A p63.2
Gag
Pol
Env
ΔC p63.2
Gag
Pol
Env
B
p63.2
WT p63.2
W390A p63.2
ΔC p63.2
Transfection
HEK 293T cells
Supernatant
RT activity
(2 dpi)
63.2 MLV
WT MLV
W390A MLV
ΔC MLV
Infection
MOI 0.215
NIH 3T3 cells
Supernatant
RT activity
(1. 2. 3. 4. 5 and 8 dpi)
C
Transfected HEK 293T cells
RTU / mL
300
250
200
150
100
50
0
*
ns
ns
p63.2
WT p63.2
W390A p63.2
ΔC p63.2
D
MLV-infected NIH 3T3 cells
RTU / mL
25000
20000
15000
10000
5000
0
1
2
3
4
5
6
7
8
9
Days post-infection (dpi)
63.2 MLV
WT MLV
W390A MLV
ΔC MLV

```
ΔC      GTGAAGGCTGCCGACCCCGGG---------------------------------------   20
WT      GTGAAGGCTGCCGACCCCGGGGGTGGACCATCCTCTAGACTGACTTGGCGCGTTCAACGC   60
W390A   GTGAAGGCTGCCGACCCCGGGGGTGGACCATCCTCTAGACTGACTGCACGCGTTCAACGC   60
aa       V  K  A  A  D  P  G  G  G  P  S  S  R  L  T  W  R  V  Q  R

ΔC      ------------------------------------------TAATCATGGCTCGTTCTA   39
WT      TCTCAAAACCCCTTAAAAATAAGGTTAACCCGCGAGGCCCCCTAATCATGGCTCGTTCTA   120
W390A   TCTCAAAACCCCTTAAAAATAAGGTTAACCCGCGAGGCCCCCTAATCATGGCTCGTTCTA   120
aa       S  Q  N  P  L  K  I  R  L  T  R  E  A  P  *

ΔC      CATTGAGCAAACCGCTTAAAAACAAAGTAAACCCACGAGGTCCGTTGATTCCCTTAATTC   99
WT      CATTGAGCAAACCGCTTAAAAACAAAGTAAACCCACGAGGTCCGTTGATTCCCTTAATTC   180
W390A   CATTGAGCAAACCGCTTAAAAACAAAGTAAACCCACGAGGTCCGTTGATTCCCTTAATTC   180
aa

ΔC      TTCTGATGCTCAGAGGGGTCAGT      122
WT      TTCTGATGCTCAGAGGGGTCAGT      203
W390A   TTCTGATGCTCAGAGGGGTCAGT      203
aa
```

```
U87552.1     CGTAAAGGCGGCCGACACCGAGAGTGGACCATCCTCTGGACGGACATGGCGCGTTCAACGCTCTCAAA
63.2 #18     CGTGAAGGCTGCCGACCCCGGGGGTGGACCATCCTCTAGACTGACATGGCGCGTTCAACGCTCTCAAA
WT #19       CGTGAAGGCTGCCGACCCCGGGGGTGGACCATCCTCTAGACTGACTTGGCGCGTTCAACGCTCTCAAA
W390A #20    CGTAAGGGCTGCCGACCCCGGGGGTGGACCATCCTCTAGACTGACTGCACGCGTACAACGCTCTAAAA
W390A #8     CGTGAAGGCTGCCGACCCCGGGGGTGGACCATCCTCTAGACTGACTGCACGCGTTCAACGCTCTCAAA
W390A #9     CGTGAAGGCTGCCGACCCCGGGGGTGGACCATCCTCTAGACTGACTGCACGCGTTCAACGCTCTCAAA
W390A #10    CGTGAAGGCTGCCGACCCCGGGGGTGGACCATCCTCTAGACTGACTGCACGCGTTCAACGCTCTCAAA
```

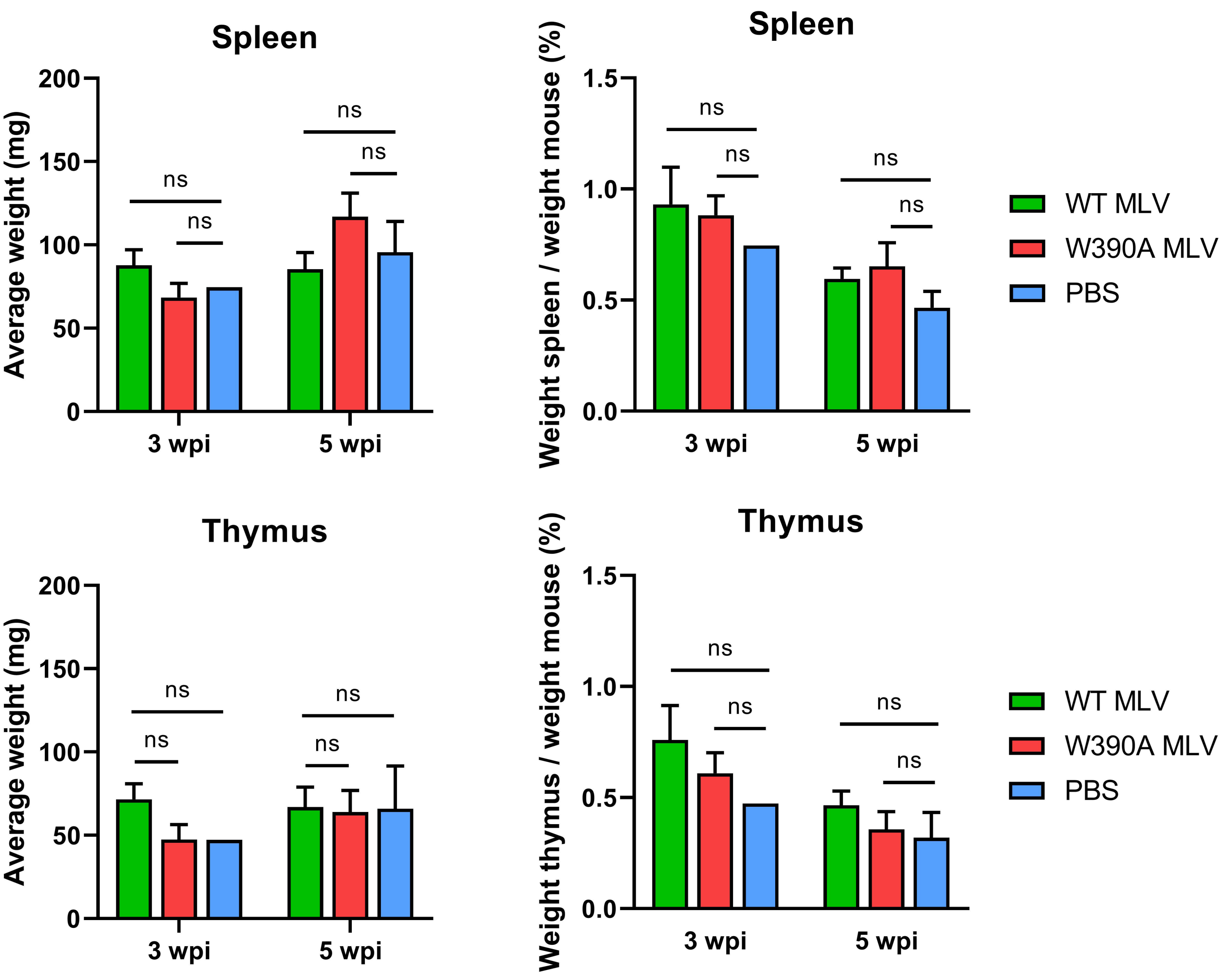

Spleen
Average weight (mg)
200
150
100
50
0
3 wpi
5 wpi
ns
ns
ns
ns
Spleen
Weight spleen / weight mouse (%)
1.5
1.0
0.5
0.0
3 wpi
5 wpi
ns
ns
ns
ns
WT MLV
W390A MLV
PBS
Thymus
Average weight (mg)
200
150
100
50
0
3 wpi
5 wpi
ns
ns
ns
ns
Thymus
Weight thymus / weight mouse (%)
1.5
1.0
0.5
0.0
3 wpi
5 wpi
ns
ns
ns
ns
WT MLV
W390A MLV
PBS

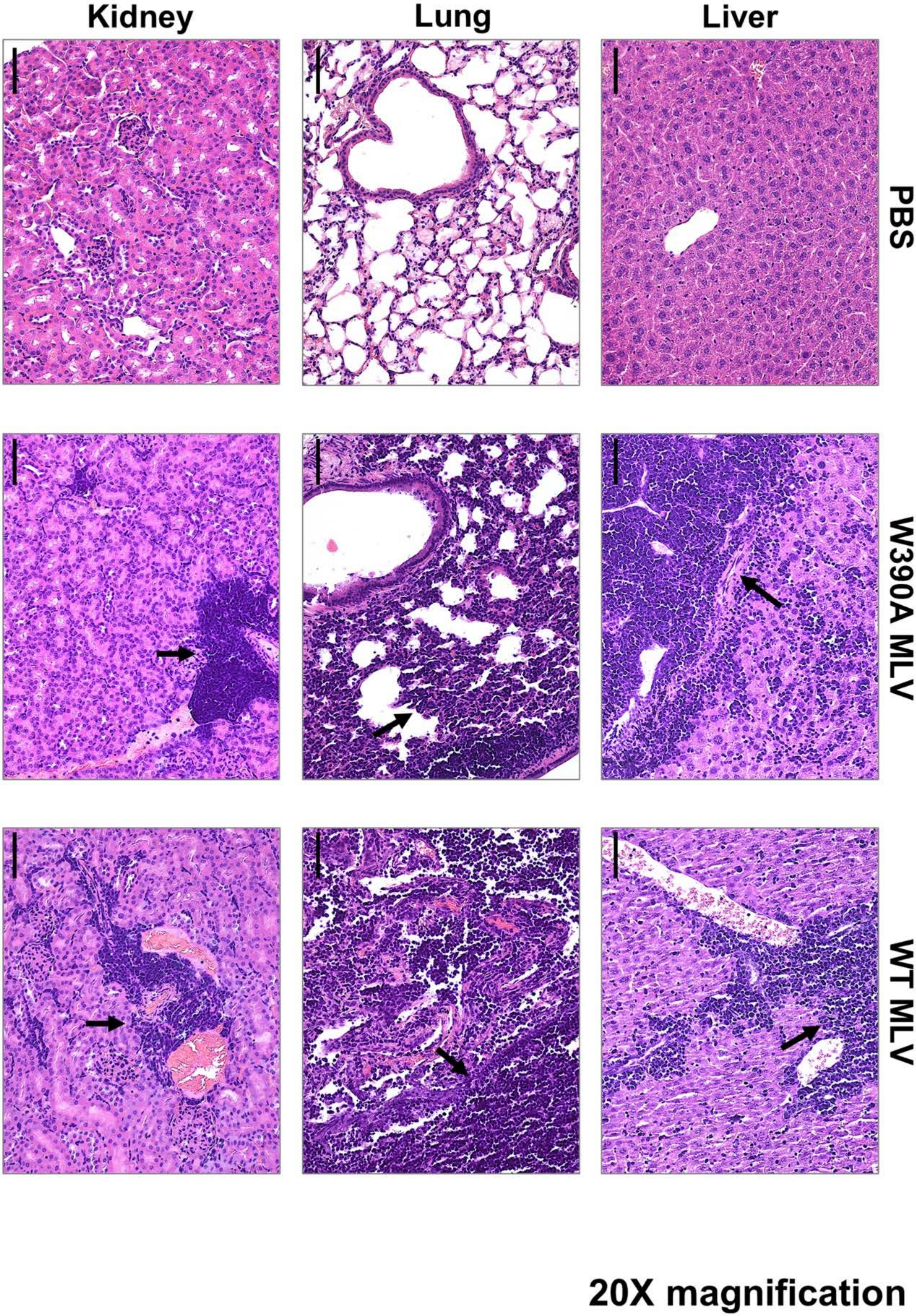
Kidney
Lung
Liver
PBS
W390A MLV
WT MLV
20X magnification

A

Clone #	WT MLV	W390A MLV	Clone #	WT MLV	W390A MLV
**1**	613	196	**17**	7	2
**2**	328	87	**18**	5	-
**3**	82	49	**19**	5	-
**4**	54	20	**20**	4	-
**5**	41	13	**21**	4	-
**6**	31	10	**22**	4	-
**7**	26	8	**23**	4	-
**8**	21	6	**24**	4	-
**9**	19	5	**25**	4	-
**10**	16	5	**26**	2	-
**11**	15	4	**27**	2	-
**12**	14	4	**28**	2	-
**13**	12	4	**29**	2	-
**14**	12	2	**30**	2	-
**15**	9	2	**31**	2	-
**16**	8	2	**32**	2	-

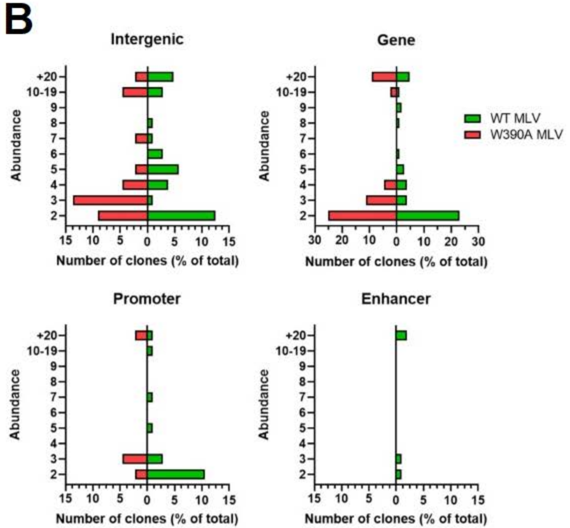

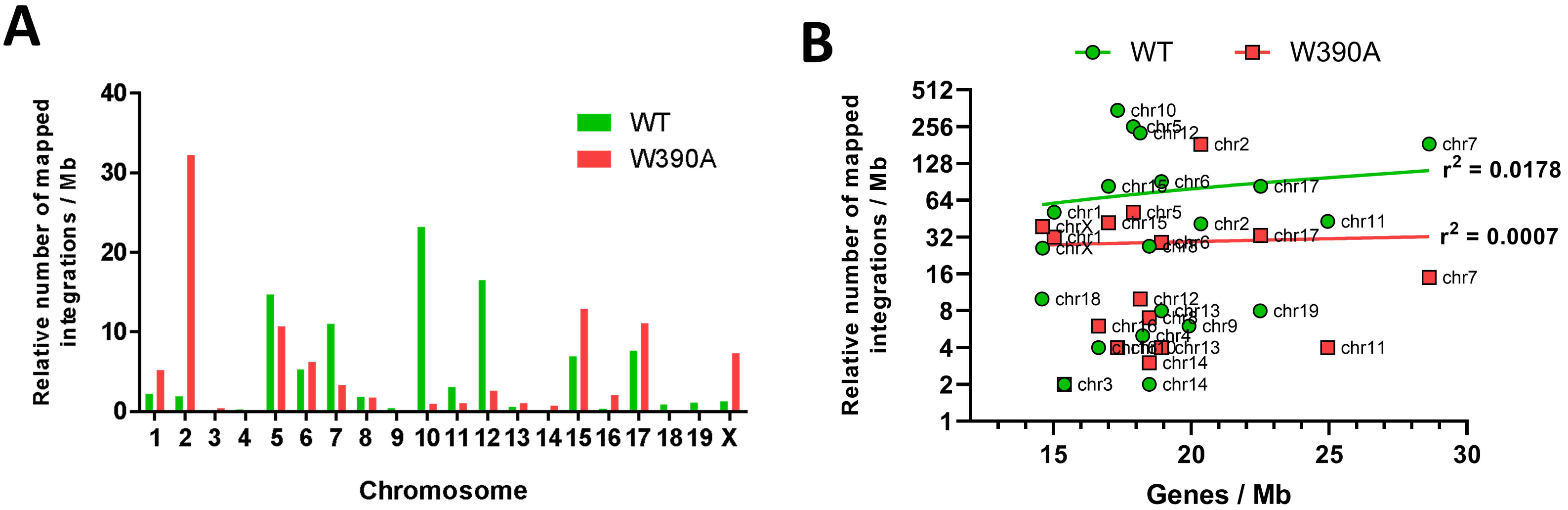
A
Relative number of mapped integrations / Mb
40
30
20
10
0
WT
W390A
1
2
3
4
5
6
7
8
9
10
11
12
13
14
15
16
17
18
19
X
Chromosome
B
WT
W390A
Relative number of mapped integrations / Mb
512
256
128
64
32
16
8
4
2
1
15
20
25
30
Genes / Mb
r² = 0.0178
r² = 0.0007
chr10
chr5
chr12
chr2
chr7
chr15
chr6
chr17
chr1
chrX
chr11
chr18
chr13
chr19
chr8
chr16
chr9
chr4
chr14
chr3

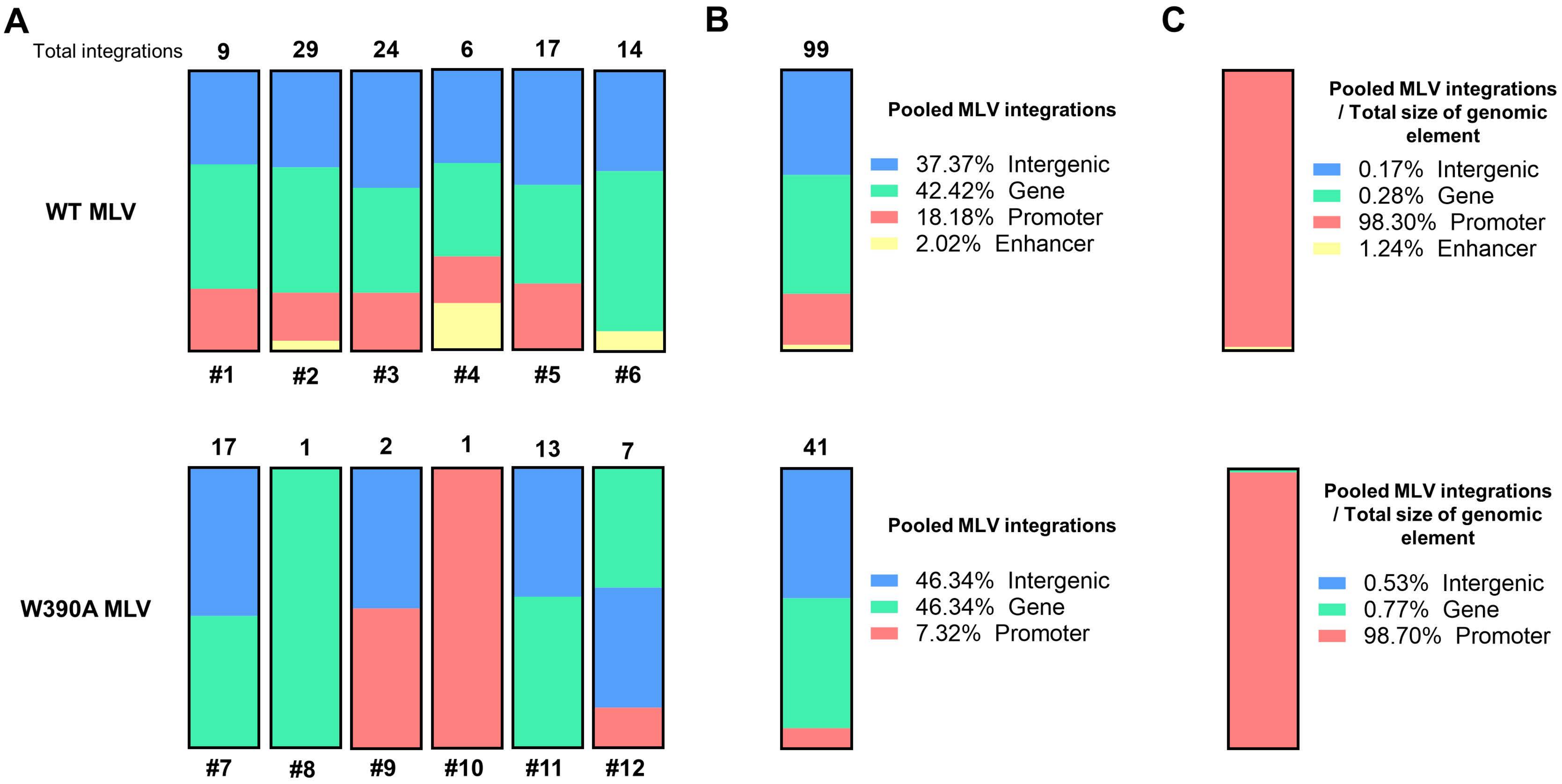
A
Total integrations
9
29
24
6
17
14
WT MLV
#1
#2
#3
#4
#5
#6
17
1
2
1
13
7
W390A MLV
#7
#8
#9
#10
#11
#12
B
99
Pooled MLV integrations
37.37% Intergenic
42.42% Gene
18.18% Promoter
2.02% Enhancer
41
Pooled MLV integrations
46.34% Intergenic
46.34% Gene
7.32% Promoter
C
Pooled MLV integrations / Total size of genomic element
0.17% Intergenic
0.28% Gene
98.30% Promoter
1.24% Enhancer
Pooled MLV integrations / Total size of genomic element
0.53% Intergenic
0.77% Gene
98.70% Promoter

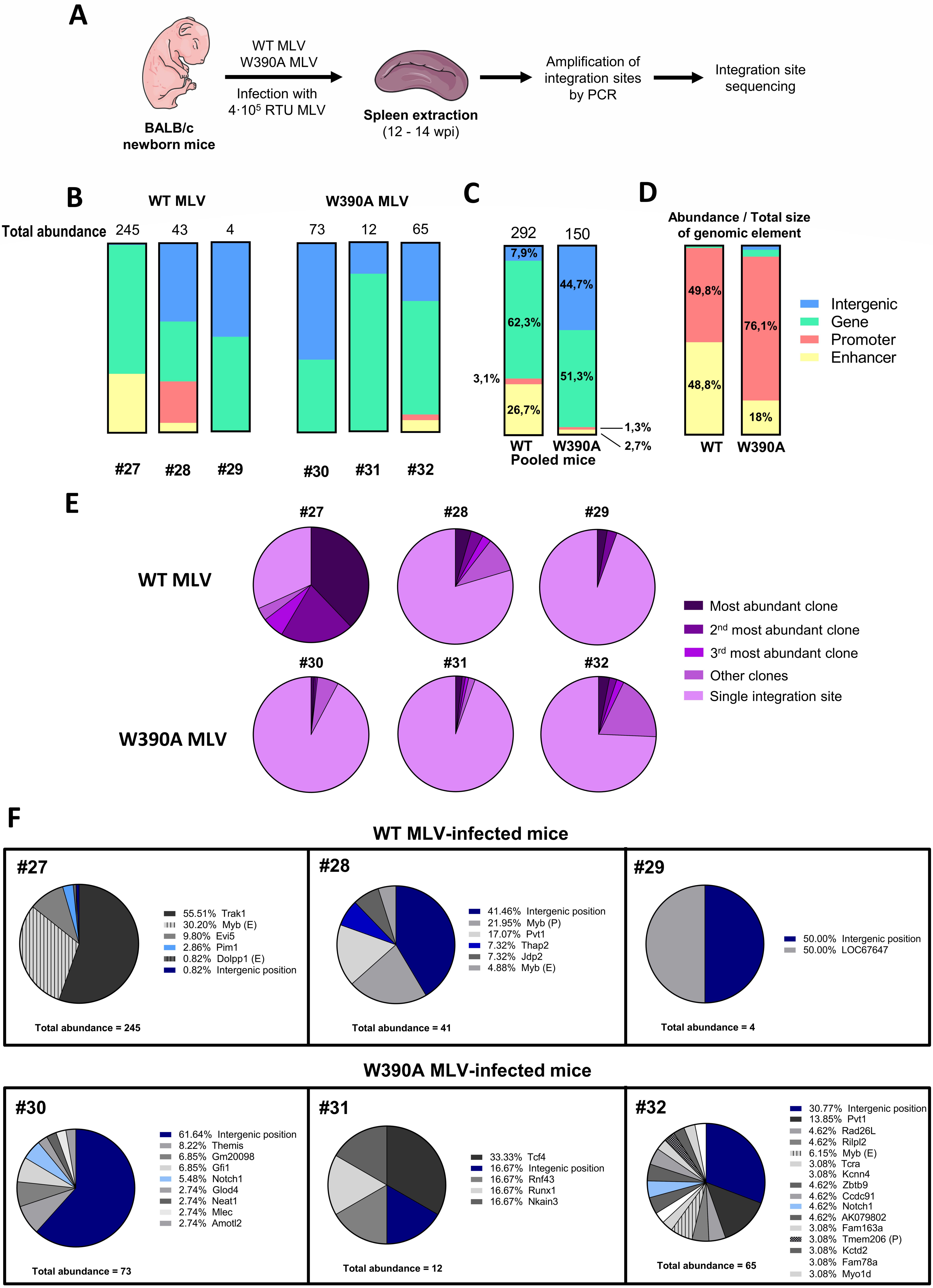
A
WT MLV
W390A MLV
Infection with
4·10⁵ RTU MLV
BALB/c
newborn mice
Spleen extraction
(12 - 14 wpi)
Amplification of
integration sites
by PCR
Integration site
sequencing
B
WT MLV
W390A MLV
Total abundance 245 43 4 73 12 65
#27 #28 #29 #30 #31 #32
C
292 150
7,9%
62,3%
3,1%
26,7%
44,7%
51,3%
1,3%
2,7%
WT W390A
Pooled mice
D
Abundance / Total size
of genomic element
49,8%
48,8%
76,1%
18%
WT W390A
Intergenic
Gene
Promoter
Enhancer
E
#27 #28 #29
WT MLV
#30 #31 #32
W390A MLV
Most abundant clone
2nd most abundant clone
3rd most abundant clone
Other clones
Single integration site
F
WT MLV-infected mice
#27
55.51% Trak1
30.20% Myb (E)
9.80% Evi5
2.86% Pim1
0.82% Dolpp1 (E)
0.82% Intergenic position
Total abundance = 245
#28
41.46% Intergenic position
21.95% Myb (P)
17.07% Pvt1
7.32% Thap2
7.32% Jdp2
4.88% Myb (E)
Total abundance = 41
#29
50.00% Intergenic position
50.00% LOC67647
Total abundance = 4
W390A MLV-infected mice
#30
61.64% Intergenic position
8.22% Themis
6.85% Gm20098
6.85% Gfi1
5.48% Notch1
2.74% Glod4
2.74% Neat1
2.74% Mlec
2.74% Amotl2
Total abundance = 73
#31
33.33% Tcf4
16.67% Integenic position
16.67% Rnf43
16.67% Runx1
16.67% Nkain3
Total abundance = 12
#32
30.77% Intergenic position
13.85% Pvt1
4.62% Rad26L
4.62% Rilpl2
6.15% Myb (E)
3.08% Tcra
3.08% Kcnn4
4.62% Zbtb9
4.62% Ccdc91
4.62% Notch1
4.62% AK079802
3.08% Fam163a
3.08% Tmem206 (P)
3.08% Kctd2
3.08% Fam78a
3.08% Myo1d
Total abundance = 65

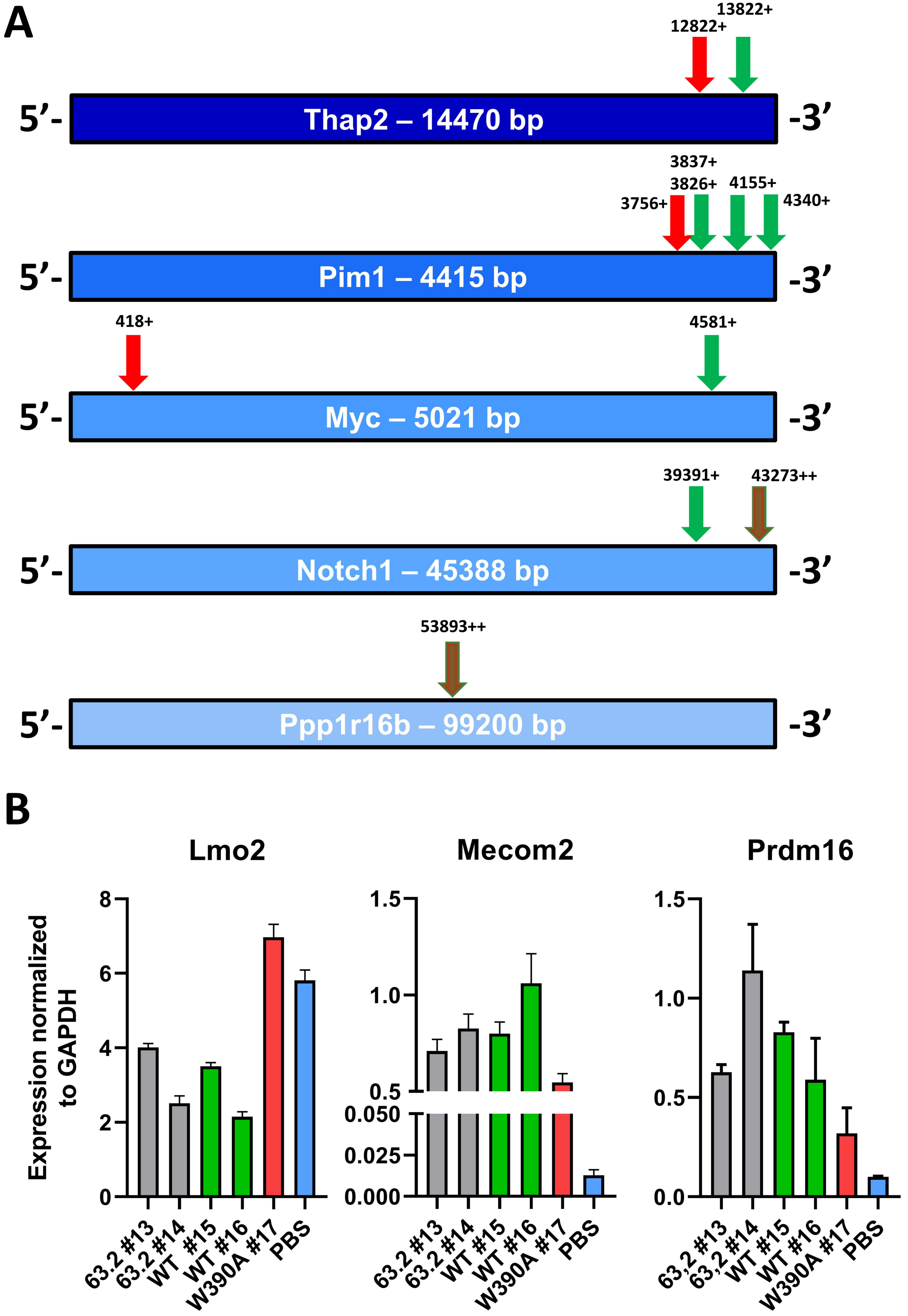
A
13822+
12822+
5′-
Thap2 – 14470 bp
-3′
3837+
3826+
4155+
4340+
3756+
5′-
Pim1 – 4415 bp
-3′
418+
4581+
5′-
Myc – 5021 bp
-3′
39391+
43273++
5′-
Notch1 – 45388 bp
-3′
53893++
5′-
Ppp1r16b – 99200 bp
-3′
B
Lmo2
Mecom2
Prdm16
Expression normalized to GAPDH
63.2 #13
63.2 #14
WT #15
WT #16
W390A #17
PBS
63,2 #13
63,2 #14
